# The bees of the family Halictidae (Hymenoptera) described by Ferdinand Morawitz from the collection of Aleksey Fedtschenko

**DOI:** 10.3897/zookeys.994.58441

**Published:** 2020-11-17

**Authors:** Yulia V. Astafurova, Maxim Yu. Proshchalykin

**Affiliations:** 1 Zoological Institute, Russian Academy of Sciences, Universitetskaya Nab., 1, Saint Petersburg 199034, Russia Zoological Institute, Russian Academy of Sciences Saint Petersburg Russia; 2 Federal Scientific Centre for East Asian Terrestrial Biodiversity, Far Eastern Branch of Russian Academy of Sciences, Vladivostok 690022, Russia Far Eastern Branch, Russian Academy of Sciences Vladivostok Russia

**Keywords:** Anthophila, Apiformes, lectotypes, Palaearctic Region, taxonomy

## Abstract

The type specimens of the family Halictidae, described by Ferdinand Morawitz from the collection of Aleksey Fedtschenko deposited in the Zoological Museum of the Moscow State University and in the Zoological Institute, Russian Academy of Sciences, St. Petersburg (Russia), are critically reviewed. Precise information with illustrations of types for 43 taxa is provided. Lectotypes are here designated for the following seven nominal taxa: *Halictus aprilinus* Morawitz, 1876, *H. cingulatus* Morawitz, 1876, *H. laevinodis* Morawitz, 1876, *H. limbellus* Morawitz, 1876, *H. nasica* Morawitz, 1876, *H. rhynchites* Morawitz, 1876 and *H. vulgaris* Morawitz, 1876.

## Introduction

More than 140 years ago (1876), the second part of Ferdinand Morawitz’s critical study on the bees collected by the Aleksey Fedtschenko 1869–1871 Expeditions in “Turkestan” was published. In the prior volume, “Apidae genuinae” (1875), Morawitz treated a total of 255 species of numerous genera, of which many species were described as new. In this second part, “Andrenidae” (1876), the remaining bees were dealt with, including the species of the difficult genera *Andrena*, *Halictus* and *Hylaeus*, totalling 183 species (Pesenko and Astafurova 2003). The species treatments are of a high professional standard, the localities are precisely documented (A. Fedtschenko 1871, O. Fedtschenko 1874, Baker 2004, Kuhlmann 2005, Dathe and Proshchalykin 2017) and the type series have been carefully conserved over a long period, generally in the collections of the Zoological Museum of the Moscow State University, Moscow, Russia (ZMMU) and in the Zoological Institute of the Russian Academy of Sciences, St. Petersburg, Russia (ZISP). To this day, these remain some of the most important manuscripts on bees of this region.

The entomological literature uses various, often obscure, terms and names for Central Asian regions and countries. The term “Turkestan” has a particularly special use in entomology, widely adopted by Morawitz (1875, 1876), Dalla Torre (1896), Meade-Waldo (1923) and in other fundamental papers, regardless of its imprecise assignment to countries (Proshchalykin and Dathe 2018).

The territory of Central Asia, described as the “Western Regions” (Xi Yui) in Chinese sources, was referred to in the Russian and European historiography of the 18^th^ and early 19^th^ Centuries as Lesser Bukharia, as opposed to Greater Bukharia, where the Bukhara Khanate was situated. In Europe, these lands came to be referred to in the 18^th^ and 19^th^ Centuries as Turkestan, i.e. “the Land of Turks,” which was the original Iranian name for the territory east of Fergana and Bukhara where nomadic Turkic tribes roamed. Subsequently, when the Turkic tribes occupied the enormous territory from the Caspian Sea to Lop Nor, the name Turkestan acquired a new meaning, so broad that it was deemed necessary to distinguish such areas as Western—Bukhara, or Russian Turkestan – and Eastern or Chinese Turkestan (Murzayev 1957). According to current views, Fedtschenko’s “Turkestan” comprises the countries Uzbekistan, Tajikistan, Kyrgyzstan and southern parts of Kazakhstan.

The family Halictidae is represented in Morawitz’s 1876 publication by four genera (*Nomioides* Schenck, 1866, *Halictus* Latreille, 1804, *Sphecodes* Latreille, 1804 and *Nomia* Latreille, 1804), comprising 73 species (Nos. 327–399). Only 30 species were previously known, while the remaining 43 were newly described (*Nomioides* – 2 species; *Halictus* – 36, *Sphecodes* – 2, *Nomia* – 2) (Table 1). Few of these taxa were mentioned in subsequent publications, remaining enigmatic for decades. Since the 1930s, V. Popov [ZISP] based his studies on the taxonomy and ecology of the Central Asian bee fauna using these collections. Popov involved other mellitologists in his research, so that specimens also reached specialists in other European museums. For example, P. Blüthgen took part in such work on *Halictus* and published several taxonomic papers, based on the Fedtschenko collection (Blüthgen 1931a, 1934a, 1955, Popov 1935).

**Table 1.** The nominal taxa of Fedtschenko’s Halictidae, described by F. Morawitz, and their current status.

N	Species name	Sex	Current status	Depositaries of types
1.	*Halictus albitarsis*	♀	Homonym	LT (ZMMU); PLT (ZISP/ZMMU)
2.	*Halictus annulipes*	♀	Valid	LT (ZMMU); PLT (ZISP/ZMMU)
3.	*Halictus aprilinus*	♀	Valid	LT, PLT (ZMMU)
4.	*Halictus atomarius*	♀	Valid	LT, PLT (ZISP)
5.	*Halictus cariniventris*	♂	Valid	LT, PLT (ZMMU)
6.	*Halictus cingulatus*	♀	Valid	LT (ZMMU); PLT (ZISP/ZMMU)
7.	*Halictus croceipes*	♀, ♂	Valid	LT (ZMMU); PLT (ZISP/ZMMU)
8.	*Halictus desertorum*	♀	Valid	LT (ZMMU)
9.	*Halictus determinatus*	♀	Homonym	LT (ZMMU); PLT (ZISP/ZMMU)
10.	*Halictus equestris*	♀	Valid	LT (ZMMU)
11.	*Halictus ferghanicus*	♂	Synonym	LT (ZMMU); PLT (ZISP)
12.	*Halictus fucosus*	♂	Synonym	HT (ZMMU)
13.	*Halictus fulvitarsis*	♂	Valid	HT (ZMMU)
14.	*Halictus funerarius*	♀	Valid	LT (ZISP); PLT (ZISP/ZMMU)
15.	*Halictus fuscicollis*	♀	Valid	LT (ZMMU); PLT (ZISP)
16.	*Halictus hyalinipennis*	♀, ♂	Valid	LT (ZISP); PLT (ZISP/ZMMU)
17.	*Halictus laevinodis*	♀	Valid	LT (ZISP); PLT (ZMMU)
18.	*Halictus limbellus*	♀	Valid	LT (ZMMU); PLT (ZISP/ZMMU)
19.	*Halictus longirostris*	♀, ♂	Valid	LT (ZMMU); PLT (ZISP/ZMMU)
20.	*Halictus maculipes*	♀	Valid	LT (ZMMU)
21.	*Halictus melanarius*	♂	Valid	HT (ZMMU)
22.	*Halictus minor*	♀	Valid	LT (ZISP); PLT (ZISP/ZMMU)
23.	*Halictus modernus*	♀	Valid	HT (ZMMU)
24.	*Halictus nasica*	♀, ♂	Valid	LT (ZMMU); PLT (ZISP/ZMMU)
25.	*Halictus nigrilabris*	♂	Valid	LT (ZMMU); PLT (ZISP)
26.	*Halictus nigripes*	♂	Homonym	LT (ZMMU); PLT (ZISP/ZMMU)
27.	*Halictus obscuratus*	♀	Valid	LT (ZMMU); PLT (ZISP/ZMMU)
28.	*Halictus palustris*	♀	Valid	LT (ZMMU); PLT (ZISP)
29.	*Halictus pectoralis*	♀	Homonym	HT (ZMMU)
30.	*Halictus picipes*	♀	Valid	LT (ZMMU); PLT (ZISP/ZMMU)
31.	*Halictus rhynchites*	♀, ♂	Valid	LT (ZMMU); PLT (ZISP/ZMMU)
32.	*Halictus scutellaris*	♀	Valid	LT (ZMMU); PLT (ZISP/ZMMU)
33.	*Halictus sogdianus*	♀	Synonym	LT, PLT (ZMMU)
34.	*Halictus trifasciatus*	♀	Homonym	LT (ZMMU)
35.	*Halictus varipes*	♀, ♂	Synonym	LT, PLT (ZMMU)
36.	*Halictus vulgaris*	♀	Synonym	LT (ZMMU); PLT (ZISP/ZMMU)
37.	*Sphecodes nigripennis*	♀	Synonym	LT, PLT (ZMMU)
38.	*Sphecodes pectoralis*	♀	Valid	LT, PLT (ZMMU)
39.	*Sphecodes rufithorax*	♀, ♂	Synonym	LT (ZMMU); PLT (ZISP/ZMMU)
40.	*Nomia edentata*	♀, ♂	Valid	LT (ZMMU); PLT (ZISP/ZMMU)
41.	*Nomia rufescens*	♀	Valid	LT (ZMMU)
42.	*Nomioides parviceps*	♂	Valid	NT (ZISP)
43.	*Nomioides turanica*	♀, ♂	Valid	LT, PLT (ZISP/ZMMU)

Comment. LT – lectotype, PLT – paralectotype/s, NT – neotype.

The second attempt to recognise the true type material of Fedtschenko’s Halictidae was made by K. Warncke, a teacher in Dachau (Germany), who visited ZMMU from 26.03.1975 to 01.04.1975 (Dathe and Proshchalykin 2017). He worked his way through the drawers containing the Fedtschenko material and labelled specimens of all nominal taxa found there as “Lectotypen.” Thereby, certain specimens from some groups of bees (*Hylaeus*, *Andrena*, *Anthidium* etc.) received a red label with the inscription “Lectotypus Warncke 1975.” However, of all Fedtschenko’s Halictidae described by Morawitz, only five specimens received such labels: *Sphecodes nigripennis* Morawitz, 1876, *S. pectoralis* Morawitz, 1876, *S. rufithorax* Morawitz, 1876, *Nomia edentata* Morawitz, 1876 and *N. rufescens* Morawitz, 1876 (Figs 32A, 33B, 34E, 35F, 36A). All other type specimens were left without nomenclatural status labels.

In his publication on the genus *Halictus*, which appeared seven years later (Warncke 1982), only the sex and locality for each lectotype is cited, for example: “*Halictus limbellus* Mor. / ♀ Lectotypus / Samarkand.” The selected specimen is not further individually identified and data on syntypes and paralectotypes are completely missing from the text. Thus, of the twenty seven designated lectotypes, fifteen are either invalid or unnecessary (when the type series includes only the holotype) and require corrections by subsequent authors (Pesenko 1986a, Ebmer 1997, current publication).

In the 1980’s, Yu. Pesenko continued the study of Halictidae in the Fedtschenko’s collection of the ZISP and ZMMU, designating lectotypes for seven of Morawitz’s nominal taxa in the genera *Halictus*, *Nomia* and *Nomioides* (Pesenko 1983, 1984, 1986a).

As a part of a detailed types inventory of the ZISP collection, all primary types of Halictidae, including seven species described by F. Morawitz from the collection of A. Fedtschenko, are being progressively photographed and catalogued (Astafurova and Proshchalykin 2018, 2019, 2020). The present paper is the first complete, illustrated summary of all species of the family Halictidae, described by F. Morawitz from the collection of A. Fedtschenko, an invaluable reference for researchers across this region who otherwise could not easily assign names to these difficult bees.

## Materials and methods

All of the material listed below was examined for this study. In the following list, the taxa are treated in alphabetical order of the names used in the original descriptions. Each entry includes the name of the taxon in its original combination, the complete reference to the original description of the species (including the original combination and spelling of the name and the author, year and page of the description) and a list of type specimens present in the collections of the ZMMU and ZISP. The data from each label are separated by two slashes (//) . Square brackets are used for English translations and when information is added to specimen label information (e.g. geographical coordinates) or published data (e.g. current name of an old place name; affiliation to a present-day country). Photographs were made using a combination of a stereomicroscope Olympus SZX10 and a digital camera (Olympus OM-D and Canon EOS70D).

Illustrations were obtained by montaging from an image series that covers different focal planes into a single in-focus image with the Helicon Focus 6. The final illustrations were post-processed for contrast and brightness using Adobe® Photoshop® software.

The classification and current species status for *Halictus* and *Lasioglossum* follow Michener (2007) and Ascher and Pickering (2020), for *Sphecodes* follow Astafurova et al. (2018a, b, 2019), for Nomiinae follow Astafurova (2014) and for Nomioidinae follow Pesenko (1983).

## Taxonomy

### List of species

#### Subfamily *Halictinae*

**Genus *Halictus* Latreille, 1804**

##### 1. Halictus albitarsis

Taxon classificationAnimaliaHymenopteraHalictidae

Morawitz, 1876

06D27E1F-7F9E-54A9-B19F-6B73690F7A82

Figure 1

Halictus albitarsis Morawitz, 1876: 217 (key to females), 246, ♀.

###### Type locality.

Samarkand (Uzbekistan).

###### Published (original) locality.

Uzbekistan: Samarkand, Tashkent.

###### Lectotype.

♀, designated by Warncke 1982: 117, <golden circle> // 21.[III.1869] // Самаркандъ [Uzbekistan, Samarkand, 39°39'N, 66°57'E] // *Halictus albitarsis* Mor., [N]372 [handwritten by F. Morawitz] // *LasioglossumEvylaeus albitarsis* Mor., ♀ = lectotype, det A.W. Ebmer 1993 // Syntypus Lectotypus <red label> [ZMMU].

###### Paralectotypes

(12 ♀). 6 ♀, 11.[III.1871], 24., 25.[V.1871] // Ташкентъ [Tashkent] // [N]372; 1 ♀, 23.[III.1871] // Ташкентъ [Tashkent] // [N]372 // *Halictus albitarsis* Mor., F. Morawitz det. [handwritten by F. Morawitz] [ZMMU]; 1 ♀, <golden circle>, 11.[III.1871] // Ташкентъ [Tashkent] // *albitarsis* Mor., Typ. [handwritten by F. Morawitz]; 3 ♀, 11.[III.1871] // Ташкентъ [Tashkent] // к.[оллекция] Ф. Моравица [Collection of F. Morawitz] //*Halictus albitarsis* Mor. [handwritten by F. Morawitz]; 1 ♀, 11.[III.1871] // Ташкентъ [Tashkent] // Paralectotypus *Halictus albitarsis* Mor., design. Warncke, [19]82 <identical red label on each paralectotype specimens, labelled by Yu. Astafurova> [ZISP].

###### Current status.

Lasioglossum (Sphecodogastra) leucopymatum (Dalla Torre, 1896), replacement name for *Halictus albitarsis* Morawitz, 1876 (nec *Hylaeus albitarsis* Schenck, 1853, nec *Halictus albitarsis* Cresson, 1872).

###### Remarks.

The secondary designation of the lectotype by Ebmer 1995: 585 is unnecessary.

Description of male. Ebmer 1995: 585.

###### Distribution.

Kazakhstan, Turkmenistan, Uzbekistan, Afghanistan (Ebmer 1995).

**Figure 1.**
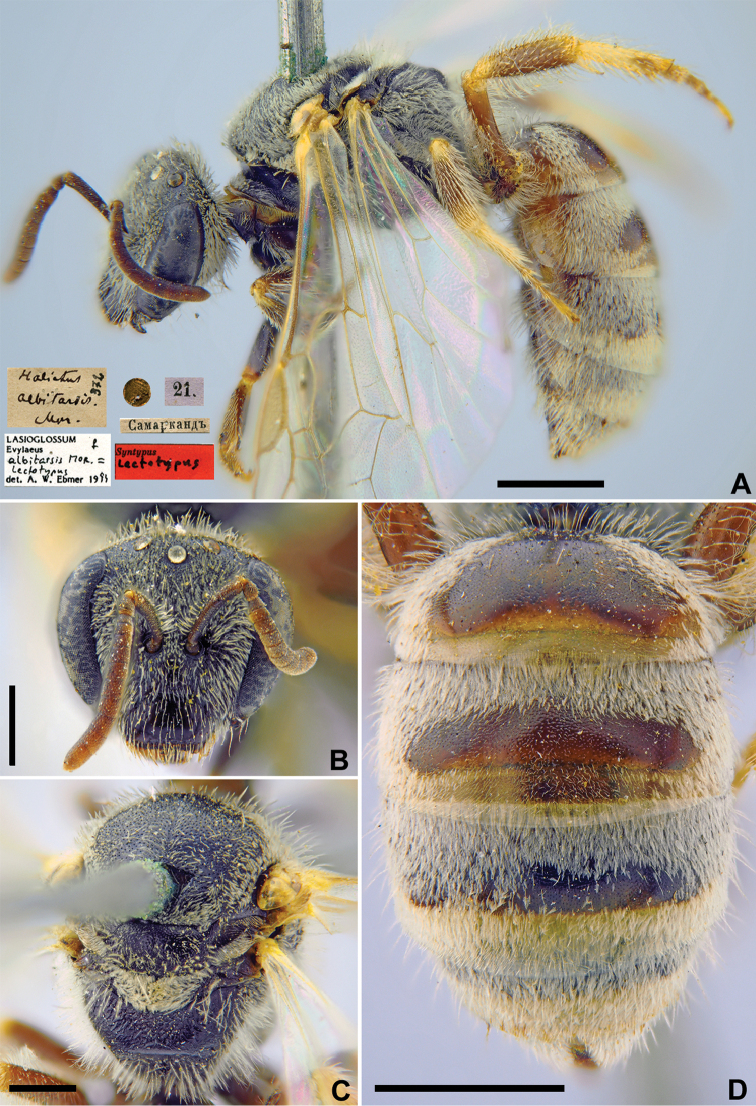
*Halictus albitarsis* Morawitz, 1876, lectotype, female **A** habitus, lateral view and labels **B** head, frontal view **C** mesosoma, dorsal view **D** metasoma, dorsal view. Scale bars: 1.0 mm (**A, D**), 0.5 mm (**B, C**).

##### 2. Halictus annulipes

Taxon classificationAnimaliaHymenopteraHalictidae

Morawitz, 1876

3DD5E1FF-FF28-5BBA-885B-0EDB0E436896

Figure 2

Halictus annulipes Morawitz, 1876: 217 (key), 221, ♀.

###### Type locality.

Karatyube Mt., 15 km S Samarkand (Uzbekistan).

###### Published (original) locality.

Uzbekistan: Samarkand, Karatyube Mt. (= 15 km S Samarkand).

###### Lectotype.

designated by Warncke 1982: 81, ♀, 20[.V.1869] // Зеравшан.[ская] дол.[ина] [Uzbekistan, Zeravshan River valley, Karatyube Mt., 39°30'N, 66°52'E] // *Halictus annulipes* Mor., [N]332 [handwritten by F. Morawitz] // F. Morawitz det. 18.7.5. // Lectotypus *Halictus annulipes* Mor. 1876, design. Warncke, 1982 <red label, labelled by Yu. Astafurova> [ZMMU].

###### Paralectotypes

(2 ♀). 1 ♀, 17. [V.1869] // Самаркандъ [Uzbekistan, Samarkand] // [N]332 // *H. annulipes* F. Morawitz det. 1875 [ZMMU]; 1 ♀, 20[.V.1869] // Зеравшан.[ская] дол.[ина] [Zeravshan River valley] // *annulipes* Mor. Typ. [handwritten by F. Morawitz] // Paralectotypus *Halictus annulipes* Mor., 1876, design. Warncke, 1982 <identical red label on each paralectotype specimen, labelled by Yu. Astafurova> [ZISP].

###### Current status.

Lasioglossum (Dialictus) annulipes (Morawitz, 1876).

###### Remarks.

Description of male. Kohl 1905: 238, as *Halictus metopias* Vachal (synonymised by Warncke 1975: 89).

###### Distribution.

Bulgaria, Armenia, Turkey, Russia (North Caucasus), Afghanistan, Iran, Tajikistan, Uzbekistan, Kazakhstan, Pakistan (Astafurova and Proshchalykin 2017, Ascher and Pickering 2020).

**Figure 2.**
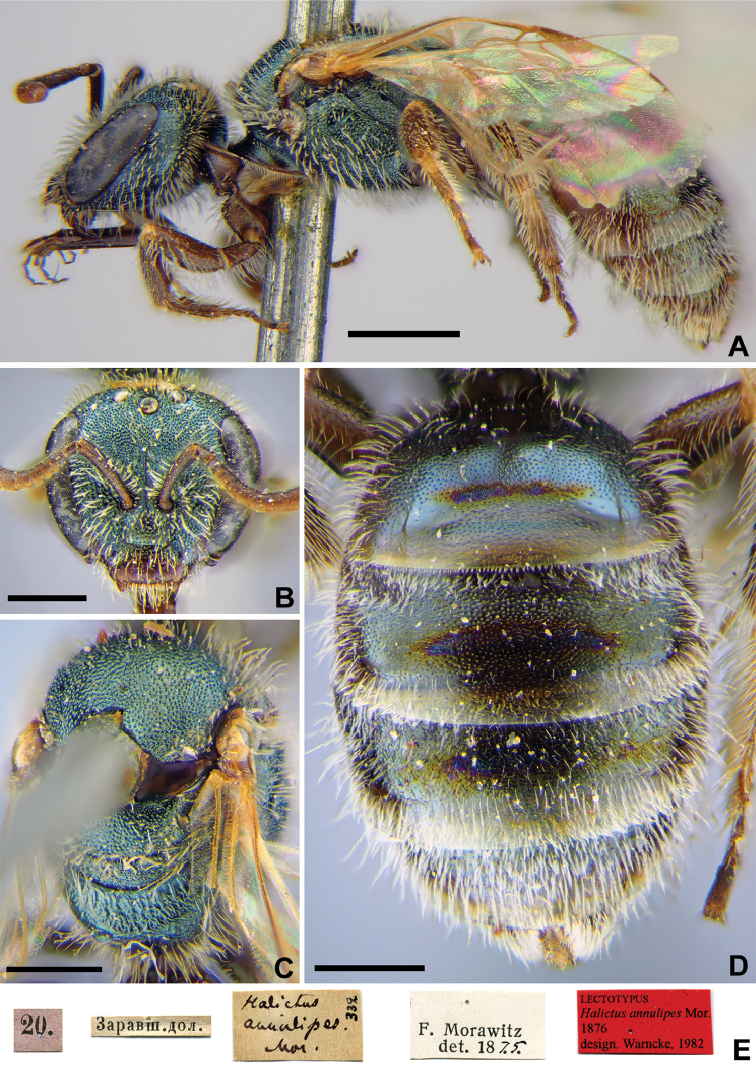
*Halictus annulipes* Morawitz, 1876, lectotype, female **A** habitus, lateral view **B** head, frontal view **C** mesosoma, dorsal view **D** metasoma, dorsal view **E** labels. Scale bars: 1.0 mm (**A**), 0.5 mm (**B–E**).

##### 3. Halictus aprilinus

Taxon classificationAnimaliaHymenopteraHalictidae

Morawitz, 1876

B05FBBBF-88E9-58A6-9110-D1C63147BFBB

Figure 3

Halictus aprilinus Morawitz, 1876: 216 (key), 228, ♀.

###### Type locality.

Kattakurgan (Uzbekistan).

###### Published (original) locality.

Uzbekistan: Katty-Kurgan.

###### Lectotype (designated here).

♀, <golden circle>, 28. [IV.1869] [Uzbekistan, Kattakurgan, 39°53'N, 66°15'E] // ♀ // *H. aprilinus* n. sp., ♀, F. Morawitz det., typus [handwritten by F. Morawitz] // Lectotypus *Halictus aprilinus* Mor., design. Astafurova et Proshchalykin, 2020 <red label> [ZMMU].

###### Paralectotypes.

3 ♀, 28. [IV.1869] // Каттыкурганъ [Kattakurgan] // [N]343 // Paralectotypus *Halictus aprilinus* Mor., design. Astafurova et Proshchalykin, 2020 <red label> [ZMMU].

###### Current status.

Lasioglossum (Sphecodogastra) aprilinum (Morawitz, 1876).

###### Remarks.

Warncke (1982: 76) did not designate the lectotype, but only wrote “Typus Mus. Moskau”.

Description of male. Blüthgen 1925a: 119, as *Halictus inexspectatus* (synonymised by Blüthgen 1931a: 212).

###### Distribution.

Southern Kazakhstan, Turkmenistan, Uzbekistan, Mongolia, China (Xinjiang) (Pesenko 2007, Murao et al. 2017).

**Figure 3.**
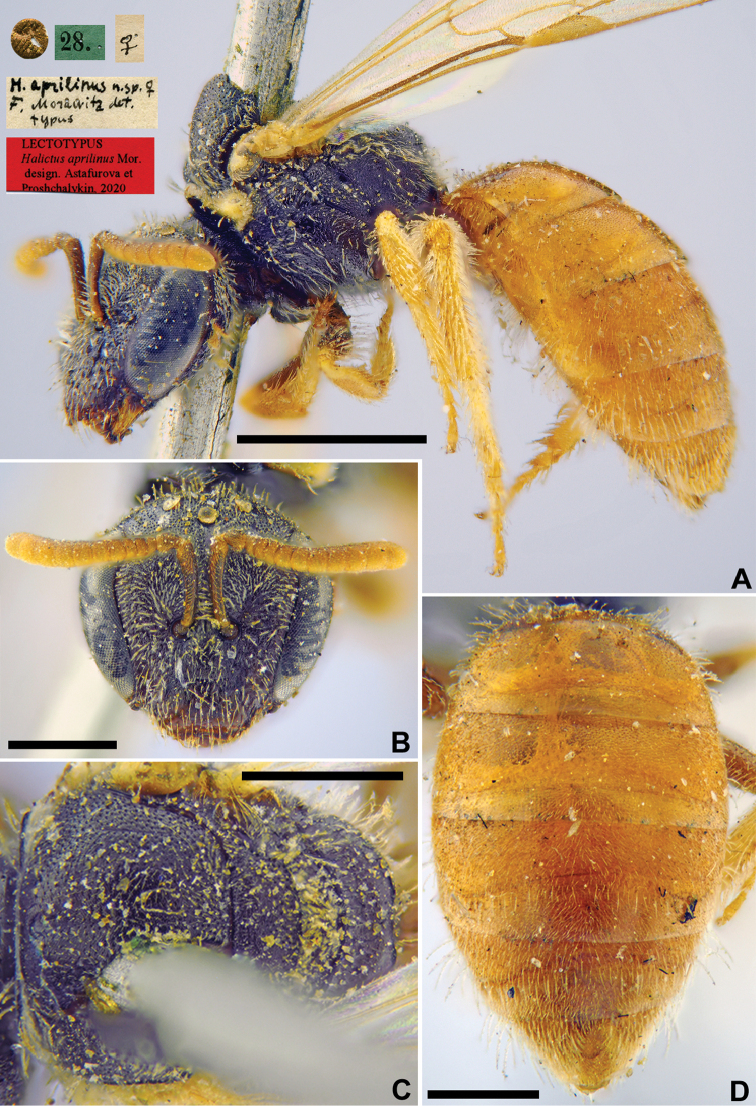
*Halictus aprilinus* Morawitz, 1876, lectotype, female **A** habitus, lateral view and labels **B** head, frontal view **C** mesosoma, dorsal view **D** metasoma, dorsal view. Scale bars: 1.0 mm (**A**), 0.5 mm (**B–D**).

##### 4. Halictus atomarius

Taxon classificationAnimaliaHymenopteraHalictidae

Morawitz, 1876

7D96A7D5-B70D-5CC6-824D-E5ADF1DA2B43

Figure (see Astafurova and Proshchalykin 2018: 11, figs 7a–e).

Halictus atomarius Morawitz, 1876: 218 (key), 254, ♀.

###### Type locality.

Tashkent (Uzbekistan).

###### Published (original) locality.

Uzbekistan: Tashkent.

###### Lectotype.

♀, designated by Ebmer 1985: 290, 8.[VIII.1870] // Ташкентъ [Uzbekistan, Tashkent, 41°18'N, 69°16'E] // *Halictus atomarius* Mor. [handwritten by F. Morawitz] // к.[оллекция] Ф. Моравица [Collection of F. Morawitz] / / Syntypus <red label> // *LasioglossumEvylaeus atomarium* (Mor.), ♀, Lectotypus, det. A.W. Ebmer 1985 // Lectotypus <red label> [ZISP].

###### Paralectotype.

1 ♀, 8.[VIII.1870] // Ташкентъ [Tashkent] // *Halictus atomarius* Mor. [handwritten by F. Morawitz] // к.[оллекция] Ф. Моравица [Collection of F. Morawitz] [ZISP].

###### Current status.

*Lasioglossum* (Evylaeus s.l.) politum atomarium (Morawitz, 1876) (subspecies status according to Ebmer 1988b: 667).

###### Remarks.

Description of male. Bytinski-Salz and Ebmer 1974: 211, as Lasioglossum politum ssp. aramaeumEbmer 1974 (synonymised by Ebmer 1985: 290).

###### Distribution.

Egypt, Palestine, Syria, Jordan, Israel, Turkey, Iran, Central Asia (Bytinski-Salz and Ebmer 1974, Warncke 1982, Pesenko 2007).

##### 5. Halictus cariniventris

Taxon classificationAnimaliaHymenopteraHalictidae

Morawitz, 1876

85C6AF01-01DF-5091-AD67-DF398449A07A

Figure 4

Halictus cariniventris Morawitz, 1876: 220 (key), 226, ♂.

###### Type locality.

Osh (Kyrgyzstan).

###### Published (original) locality.

Uzbekistan: Samarkand, Dzhyuzak [=Jizzakh], Sokh; Kyrgyzstan: Osh.

###### Lectotype.

♂, designated by Blüthgen 1955: 19, 1.[VIII.1871] // Ошъ [Kyrgyzstan, Osh, 40°32'N, 72°47'E] // *Halictus cariniventris* Mor., [N]341 [handwritten by F. Morawitz] // Lectotypus *Halictus cariniventris* Mor., design. Blüthgen <red label, labelled by Yu. Astafurova> [ZMMU].

###### Paralectotypes

(4 ♂). 2 ♂, 4., 7.[VII.1869] // Самарканд [Samarkand] // [N]341; 2 ♂, 18., 22.[VII.1870] // Джюзакъ [Dzhyuzak] // [N]341 // Paralectotypus *Halictus cariniventris*, design. Blüthgen <identical red labels on each paralectotype specimen, labelled by Yu. Astafurova> [ZMMU].

###### Current status.

Halictus (Mucoreochalictus) pollinosa cariniventris Morawitz, 1876 (subspecies status according to Ebmer 1988b: 578).

###### Remarks.

The lectotype designation by Warncke (1982: 138) is unnecessary.

Description of female: Strand 1921: 314, as Halictus cariniventris var. creticola (synonymised by Ebmer 1988b: 578).

###### Distribution.

Europe (except north), Russia (European part, except north), Turkey, Israel, Iran, Afghanistan, Pakistan, Central Asia, Mongolia, North China (Pesenko 2006a, Astafurova and Proshchalykin 2017).

**Figure 4.**
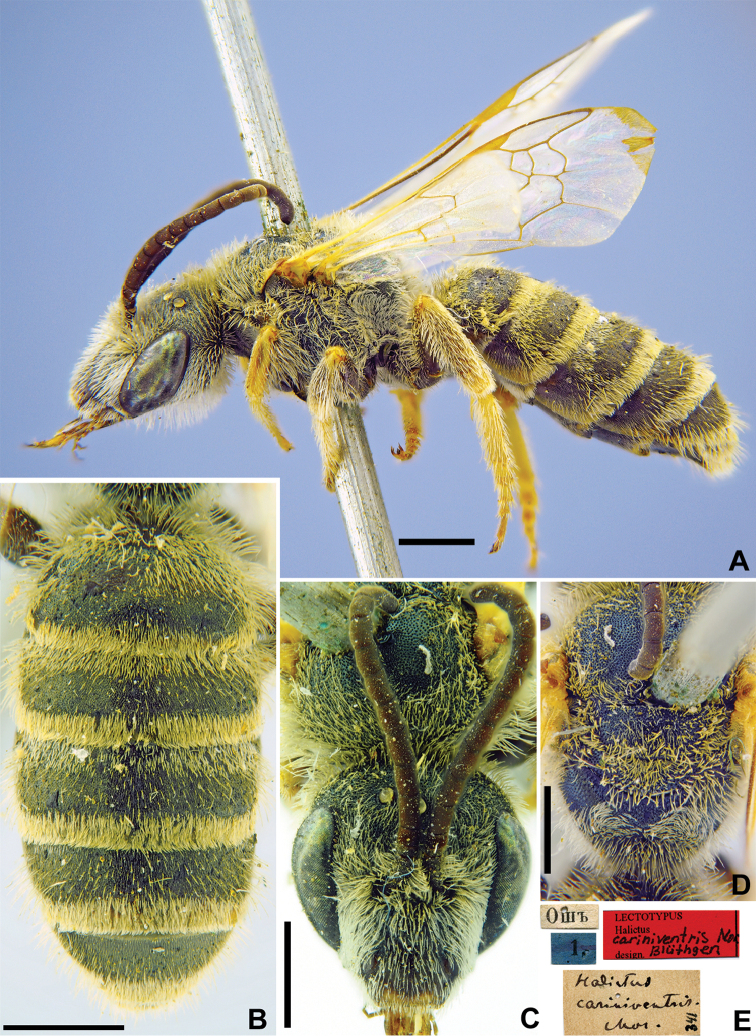
*Halictus cariniventris* Morawitz, 1876, lectotype, male **A** habitus, lateral view **B** metasoma, dorsal view **C** head, frontal view **D** mesosoma, dorsal view **E** labels. Scale bars: 1.0 mm.

##### 6. Halictus cingulatus

Taxon classificationAnimaliaHymenopteraHalictidae

Morawitz, 1876

ED601DFE-909A-5859-AE9F-EB0E15954C64

Figure 5

Halictus cingulatus Morawitz, 1876: 218 (key), 245, ♀.

###### Type locality.

Samarkand (Uzbekistan).

###### Published (original) locality.

Uzbekistan: Samarkand, Dzham [near Samarkand], Aksay [near Samarkand].

###### Lectotype (designated here).

♀, 18.[III.1869] // Самаркандъ [Uzbekistan, Samarkand, 39°39'N, 66°57'E] // *Halictus cingulatus* Mor., [N]371 [handwritten by F. Morawitz] // Lectotypus *Halictus cingulatus* Mor., design. Astafurova et Proshchalykin, 2020 <red label> [ZMMU].

###### Paralectotypes

(22 ♀). 11 ♀, 27., 28. [II. 1869], 18., 21. [V. 1869] // Самаркандъ [Samarkand] // [N]371; 1 ♀, 12.[V.1969] // Заравшан.[ская] дол.[ина] [Zeravshan River valley, Dzham] // [N]370; 1 ♀, 16.[V.1969] // Заравшан.[ская] дол.[ина] [Zeravshan River valley, Aksay] // [N]370 [ZMMU]; 1 ♀, the same labels, but 21.[V.1869] [ZMMU]; 1 ♀, <golden circle> // 18.[V.1869] // Самаркандъ [Samarkand] // *cingulatus* Mor. Typ. [handwritten by F. Morawitz]; 1 ♀, 18.[III.1869] // Самаркандъ [Samarkand] // к.[оллекция] Ф. Моравица [Collection of F. Morawitz]; 1 ♀, 20.[III.1869] // Самаркандъ [Samarkand] // к.[оллекция] Ф. Моравица [Collection of F. Morawitz] // *Halictus cingulatus* Mor., ♀, CoType, F. Morawitz det. [handwritten by F. Morawitz]; 1 ♀, 27.[III.1869] // Самаркандъ [Samarkand] // к.[оллекция] Ф. Моравица [Collection of F. Morawitz]; 1 ♀, 16.[III.1869] // Самаркандъ [Samarkand] // к.[оллекция] Ф. Моравица [Collection of F. Morawitz] // *Halictus cingulatus* Mor., ♀, F. Morawitz det. [handwritten by F. Morawitz]; 1 ♀, 30.[III.1869] // Заравшан.[ская] дол.[ина] [Zeravshan River valley, Samarkand] // *Halictus cingulatus* Mor. [handwritten by F. Morawitz]; 2 ♀, 12.[III.1869] // Заравшан.[ская] дол.[ина] [Zeravshan River valley, Samarkand] // к.[оллекция] Ф. Моравица [Collection of F. Morawitz] // Paralectotypus *Halictus cingulatus* Mor., design. Astafurova et Proshchalykin, 2020 <identical red labels on each paralectotype specimen> [ZISP].

###### Current status.

Lasioglossum (Sphecodogastra) cingulatum (Morawitz, 1876).

###### Remarks.

Male unknown.

The lectotype designation by Warncke (1982: 116) is invalid because he labelled none of the eleven females from Samarkand deposited in ZMMU.

###### Distribution.

South Kazakhstan, Central Asia, Pakistan (Ebmer 1995, Murao et al. 2017).

**Figure 5.**
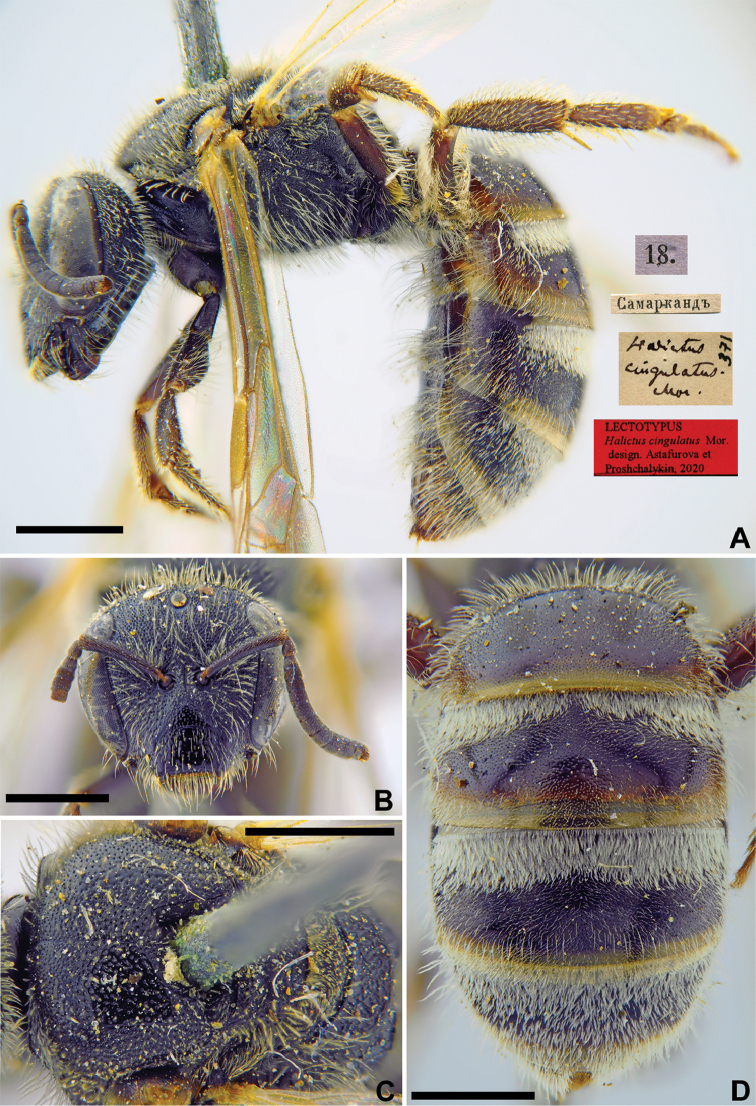
*Halictus cingulatus* Morawitz, 1876, lectotype, female **A** habitus, lateral view and labels **B** head, frontal view **C** mesosoma, dorsal view **D** metasoma, dorsal view. Scale bars: 1.0 mm.

##### 7. Halictus croceipes

Taxon classificationAnimaliaHymenopteraHalictidae

Morawitz, 1876

0C3828D5-D7F1-5756-9507-F7DD0D8AE2DF

Figure 6

Halictus croceipes Morawitz, 1876: 217 (key to ♀), 220 (key to ♂), 224, ♀, ♂.

###### Type locality.

Yeri, Sughd Province (Tajikistan).

###### Published (original) locality.

Kyrgyzstan: Taka; Uzbekistan: Karatyube [Mt., 15 km S Samarkand], Dzham Gorge, Urgut; Tajikistan: Iori.

###### Lectotype.

♀, designated by Ebmer 1997: 956, <golden circle> // 1.[VI.1869] // Заравш.[анская] дол.[ина] [Tajikistan, Zeravshan River valley, Iori (= Yeri), 39°30'N, 67°52'E] // *croceipes* Mor. Typ [handwritten by F. Morawitz] // *croceipes* Mor. Blüthgen det. 1935 // Lectotype <red label> // Lasioglossum (Evylaeus) croceipes (Mor.), ♀, Lectotypus, det. A.W. Ebmer 1974 [ZMMU].

###### Paralectotypes

(6 ♀). 1 ♀, 20.[V.1869] // Заравшан.[ская] дол.[ина] [Zeravshan River valley, Karatyube] // *croceipes* Mor., [N]338 [handwritten by F. Morawitz] // *croceipes* Mor. Blüthgen det. 1935; 3 ♀, 8.[VIII.1871] // Така [Taka] // [N]338; 1 ♀, 13.[V.1869] // Джамское ущ. [Dzham Gorge] // [N]338 [ZMMU]; 1 ♀, 1.[VI.1869] // Заравшан.[ская] дол.[ина] [Zeravshan River valley, Iori] // к.[оллекция] Ф. Моравица [Collection of F. Morawitz] // *croceipes* Mor., Blüthgen det. 1935 // Paralectotypus *Halictus croceipes* Mor., design. Ebmer 1997 <identical red labels on each paralectotype specimen, labelled by Yu. Astafurova> [ZISP].

###### Current status.

Lasioglossum (Hemihalictus) croceipes (Morawitz, 1876).

###### Remarks.

The lectotype designation by Warncke (1982: 106) is invalid because there are two females in ZMMU from the Zeravshan River valley, neither with Warncke’s lectotype label.

###### Distribution.

Turkey, Iran, Afghanistan, Central Asia, Kazakhstan (Ebmer 1997, Murao et al. 2017).

**Figure 6.**
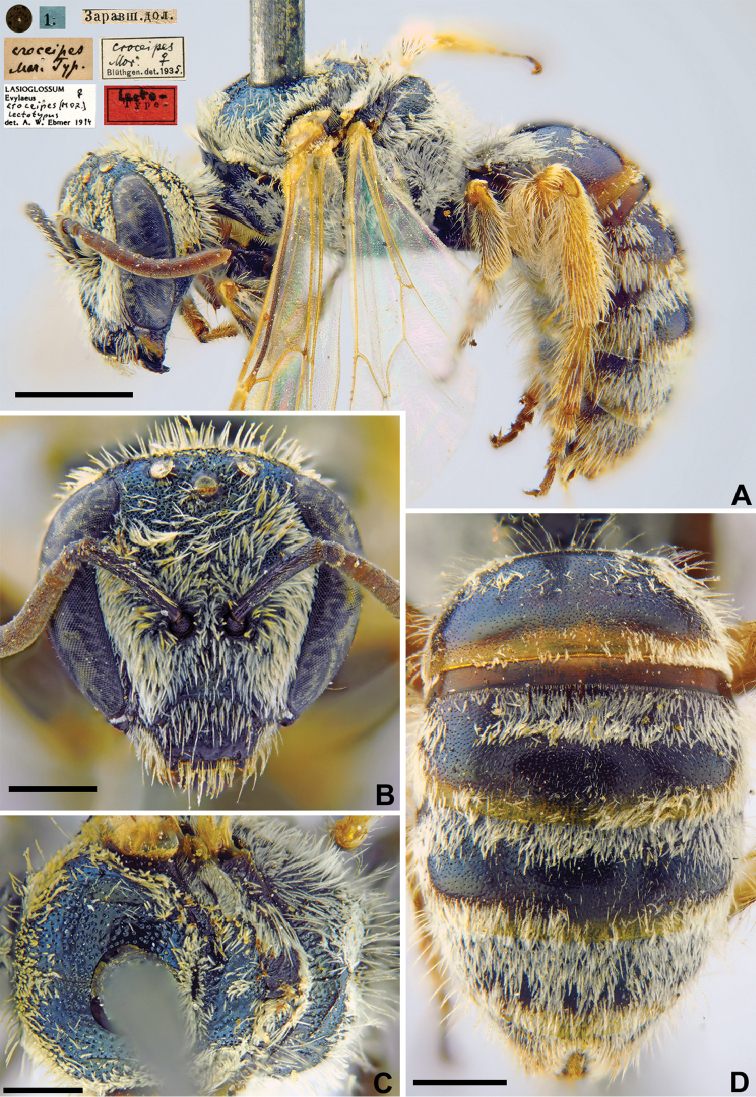
*Halictus croceipes* Morawitz, 1876, lectotype, female **A** habitus, lateral view and labels **B** head, frontal view **C** mesosoma, dorsal view **D** metasoma, dorsal view. Scale bars: 1.0 mm (**A, D**), 0.5 mm (**B, C**).

##### 8. Halictus desertorum

Taxon classificationAnimaliaHymenopteraHalictidae

Morawitz, 1876

D1065512-A6E2-5834-99CF-F239AE07EA09

Figure 7

Halictus desertorum Morawitz, 1876: 217 (key), 228, ♀.

###### Type locality.

Kattakurgan (Uzbekistan).

###### Published (original) locality.

Uzbekistan: Katty-Kurgan.

###### Lectotype.

♀, designated by Warncke 1982: 107, 28.[IV.1869] // Катты-Курганъ [Uzbekistan, Katty-Kurgan (=Kattakurgan), 39°53'N, 66°15'E] // *Halictus desertorum* Mor., [N]344 [handwritten by F. Morawitz] // Lectotypus *Halictus desertorum* Mor., design. Warncke <red label, labelled by Yu. Astafurova> [ZMMU].

###### Current status.

Halictus (Placidochalictus) desertorum Morawitz, 1876.

###### Remarks.

Description of male. Blüthgen 1929: 84, figs 9a, b.

###### Distribution.

Turkey, southern Kazakhstan, Turkmenistan, Uzbekistan, Pakistan (Blüthgen 1929, Ebmer 1988a).

**Figure 7.**
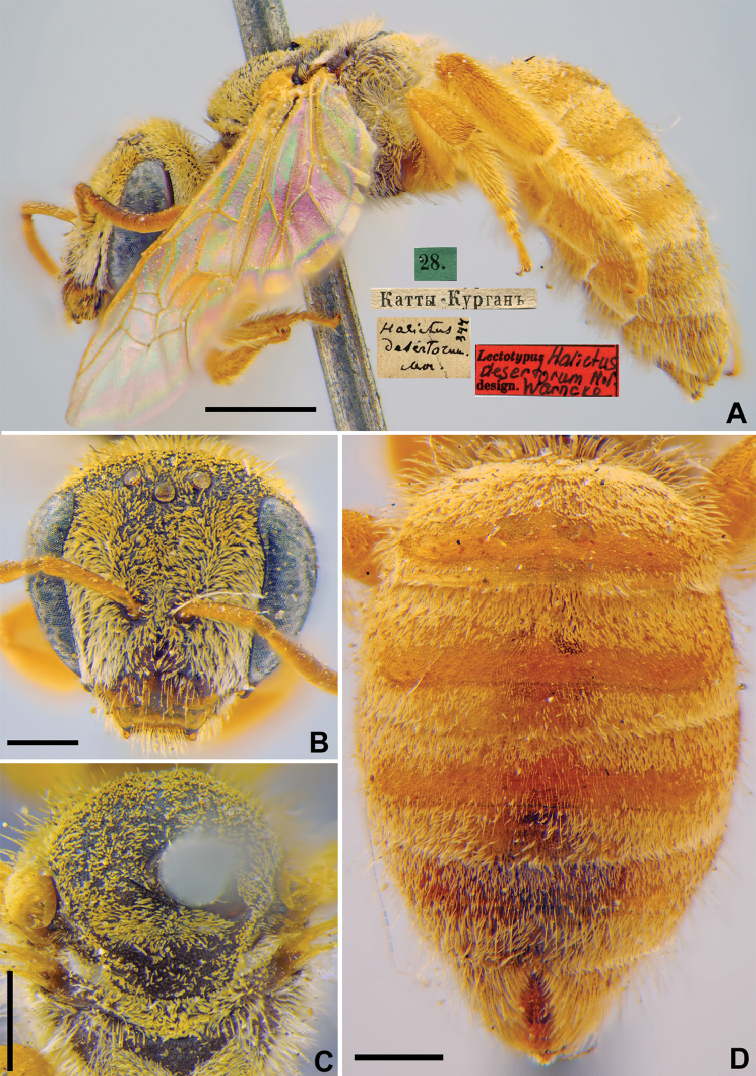
*Halictus desertorum* Morawitz, 1876, lectotype, female **A** habitus, lateral view and labels **B** head, frontal view **C** mesosoma, dorsal view **D** metasoma, dorsal view. Scale bars: 1.0 mm (**A**), 0.5 mm (**B–D**).

##### 9. Halictus determinatus

Taxon classificationAnimaliaHymenopteraHalictidae

Morawitz, 1876

23A7E390-6AFD-58F8-8C57-54164BF4725B

Figure 8

Halictus determinatus Morawitz, 1876: 217 (key), 233, ♀.

###### Type locality.

30 km SSE Samarkand, Sangu-dzhuman Pass (Uzbekistan).

###### Published (original) locality.

Uzbekistan: “On the road to Sangy-Dzhuman [pass] and the Kulbasy Mountain”.

###### Lectotype.

♀, designated by Pesenko 1984: 20, 25.[V.1869] // Сангы-Джуманъ [Uzbekistan, Sangu-dzhuman Pass, Zeravshan Ridge, 30 km SSE Samarkand, 39°27'N, 67°14'E] // *Halictus determinatus* Mor., [N]351 [handwritten by F. Morawitz] // Lectotypus *H. determinatus* Mor., ♀, design. Pesenko [1]981 <red label> [ZMMU].

###### Paralectotypes

(6 ♀). 3 ♀, the same label as in the lectotype; 1 ♀, 25.[V.1869] // Заравшан.[ская] дол.[ина] [Zeravshan River valley] // [N]351 [ZMMU]; 2 ♀, Сангы-Джуманъ [Sangu-Dzhuman] // к.[оллекция] Ф. Моравица [Collection of F. Morawitz] // *determinatus* F. Mor., ♀ [handwritten by F. Morawitz] // Paralectotypus *H. determinatus* Mor., ♀, design. Pesenko [1]981 <identical red labels on each paralectotype specimen, labelled by Yu. Pesenko> [ZISP].

###### Current status.

Halictus (Platyhalictus) determinandus Dalla Torre, 1896, replacement name for *H. determinatus* Morawitz, 1876 (nec *H. determinatus* Walker, 1871).

###### Remarks.

Description of male. Ebmer 1980: 471, Fig. 1.

###### Distribution.

A rare montane Central Asian species. Northern Afghanistan, eastern Uzbekistan, Tajikistan and Kyrgyzstan (Pesenko 2005a).

**Figure 8.**
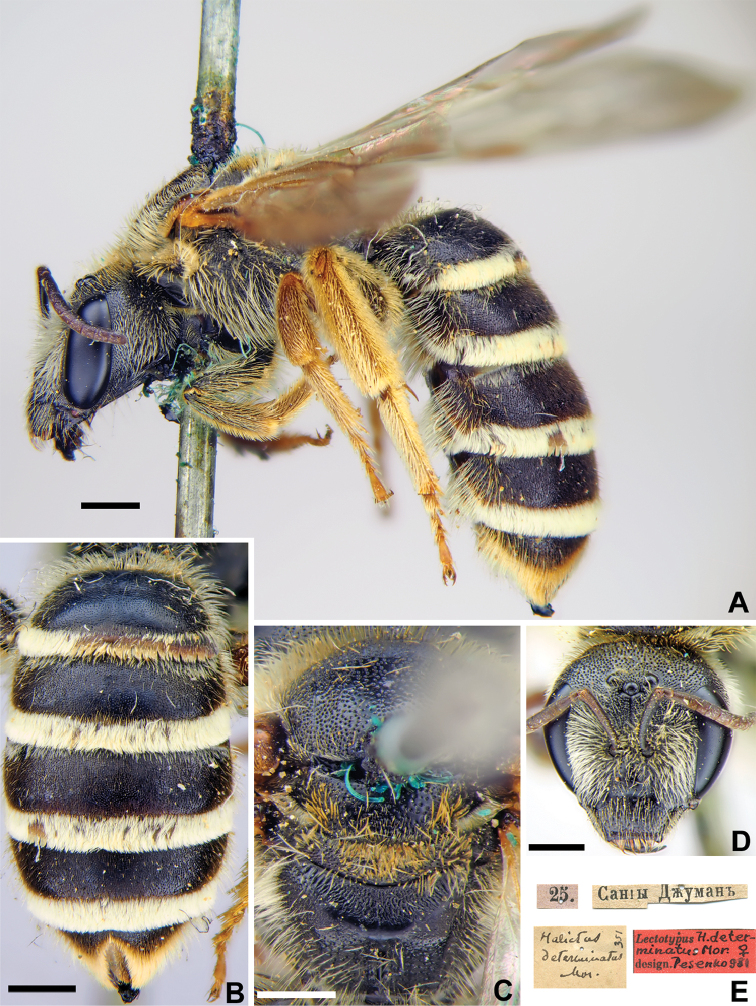
*Halictus determinatus* Morawitz, 1876, lectotype, female **A** habitus, lateral view **B** metasoma, dorsal view **C** mesosoma, dorsal view **D** head, frontal view **E** labels. Scale bars: 1.0 mm.

##### 10. Halictus equestris

Taxon classificationAnimaliaHymenopteraHalictidae

Morawitz, 1876

769169D6-863A-5767-B503-0384BF866B38

Figure 9

Halictus equestris Morawitz, 1876: 217 (key), 242, ♀.

###### Type locality.

Khozyay-Dun (Uzbekistan).

###### Published (original) locality.

Uzbekistan: Khodzhaduk [= Khozyay-Dun].

###### Lectotype.

♀, designated by Warncke 1982: 105, 21.[V.1869] // Заравшан.[ская] дол.[ина] [Uzbekistan, Zeravshan River valley, Khozyay-Dun, 39°24'N, 67°01'E] // *Halictus equestris* Mor., [N]366 [handwritten by F. Morawitz] // Lectotypus *Halictus equestris* Mor., design. Warncke [19]82 <red label> [ZMMU].

###### Current status.

Lasioglossum (Lasioglossum) equestre (Morawitz, 1876).

###### Remarks.

Description of male. Morawitz 1876: 219 (key), 243, as *Halictus ferghanicus* (synonymised by Blüthgen 1926: 391).

According to the Fedchenko list (Baker 2004: 243), the locality given in Warncke’s lectotype designation is in Uzbekistan, not Tajikistan.

###### Distribution.

Turkey, Uzbekistan, Tajikistan, Kyrgyzstan, south-eastern Kazakhstan (Pesenko 1986a, Ascher and Pickering 2020).

**Figure 9.**
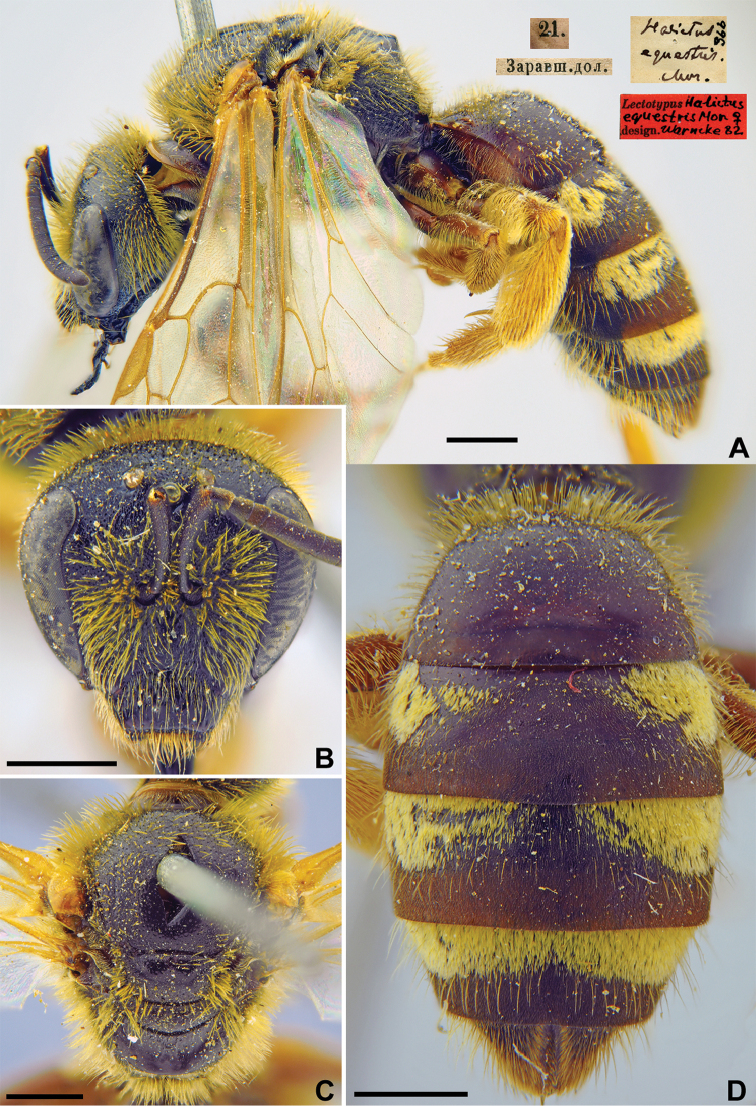
*Halictus equestris* Morawitz, 1876, lectotype, female **A** habitus, lateral view and labels **B** head, frontal view **C** mesosoma, dorsal view **D** metasoma, dorsal view. Scale bars: 1.0 mm.

##### 11. Halictus ferghanicus

Taxon classificationAnimaliaHymenopteraHalictidae

Morawitz, 1876

BD5F0E37-23E8-58FA-908F-A3558314BEE3

Figure 10

Halictus ferghanicus Morawitz, 1876: 219 (key), 243, ♂.

###### Type locality.

Shakhimardan (Uzbekistan).

###### Published (original) locality.

Uzbekistan: near Shakhimardan.

###### Lectotype.

♂, designated by Warncke 1982: 105, <golden circle> // 6.[VII.1871] // Шагимарданъ [Shakhimardan in the Uzbek enclave in the territory of Kyrgyzstan, Alai Ridge; 39°58'N, 71°47'E] // *Halictus ferghanicus* Mor., [N]367 [handwritten by F. Morawitz] // Lectotypus *Halictus ferghanicus* Mor., ♂, design. Warncke [19]82 <red label, labelled by Yu. Pesenko > [ZMMU].

###### Paralectotypes

(2 ♂). 1 ♂, <golden circle> // 2.[VII.1871] // Шагимарданъ [Shagimardan] // *ferghanicus* Mor., Typ. [handwritten by F. Morawitz]; 1 ♂, 2.[VII.1871] // Шагимарданъ [Shagimardan] // к.[оллекция] Ф. Моравица [Collection of F. Morawitz] // *Halictus ferghanicus* M. [handwritten by F. Morawitz] // *Lasioglossum equestre* Mor., Pesenko det., 1985 // Paralectotypus, *Halictus ferghanicus* Mor., design. Warncke, [19]82 <identical red labels on each paralecotype specimen, labelled by Yu. Pesenko> [ZISP].

###### Current status.

Lasioglossum (Lasioglossum) equestre (Morawitz, 1876) (synonymised by Blüthgen 1926: 391).

###### Distribution.

See *Halictus equestris* Morawitz, 1876.

**Figure 10.**
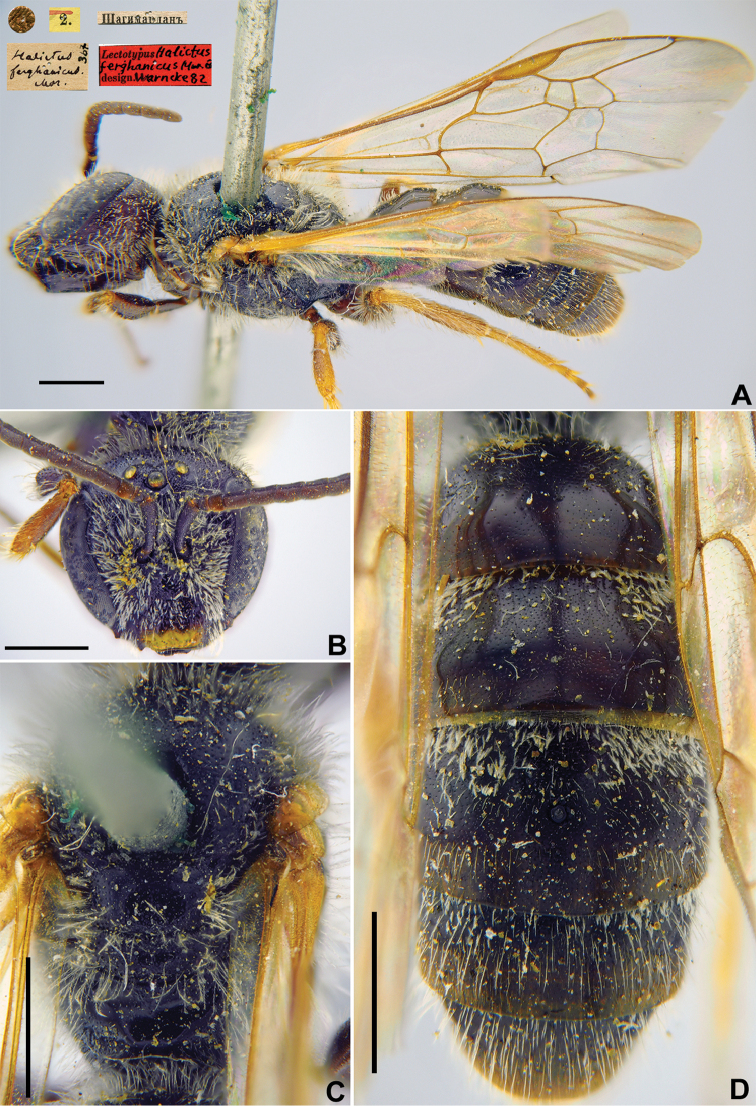
*Halictus ferghanicus* Morawitz, 1876, lectotype, male **A** habitus, lateral view and labels **B** head, frontal view **C** mesosoma, dorsal view **D** metasoma, dorsal view. Scale bars: 1.0 mm.

##### 12. Halictus fucosus

Taxon classificationAnimaliaHymenopteraHalictidae

Morawitz, 1876

E2FF2321-642E-53B7-92A4-441223EDEA8C

Figure 11

Halictus fucosus Morawitz, 1876: 219 (key), 230, ♂.

###### Type locality.

30 km SE Kozhatogai, Turkistan Province (Kazakhstan).

###### Published (original) locality.

Uzbekistan: steppe between Tashkent and Syrdarya River.

###### Holotype.

♂, 18.[V.1871] // Степь м.[ежду] С.[ыр] д.[арьей] и Т.[ашкентом] [Kazakhstan, Turkistan Province, steppe between Syrdarya River and Tashkent, 30 km SE Kozhatogai, 41°47'N, 68°23'E] // *Halictus fucosus* Mor., [N]345 [handwritten by F. Morawitz] // *Halictus senilis* Evers. v. *fucosus* Mor., ♂, P. Blüthgen det. // Holotypus <red label> [ZMMU].

###### Current status.

Halictus (Argalictus) senilis (Eversmann, 1852) (synonymised by Blüthgen 1922: 47).

###### Remarks.

The lectotype designation of Warncke (1982: 148) is unnecessary as the species was described from a single male that was directly written about by Morawitz (1876: 231).

###### Distribution.

North Africa, South and East Europe, Russia (south to Urals Mountains on the east), Caucasus, Turkey, Near East, Iraq, Iran, Afghanistan, Central Asia, Kazakhstan, Mongolia, China (Astafurova and Proshchalykin 2017).

**Figure 11.**
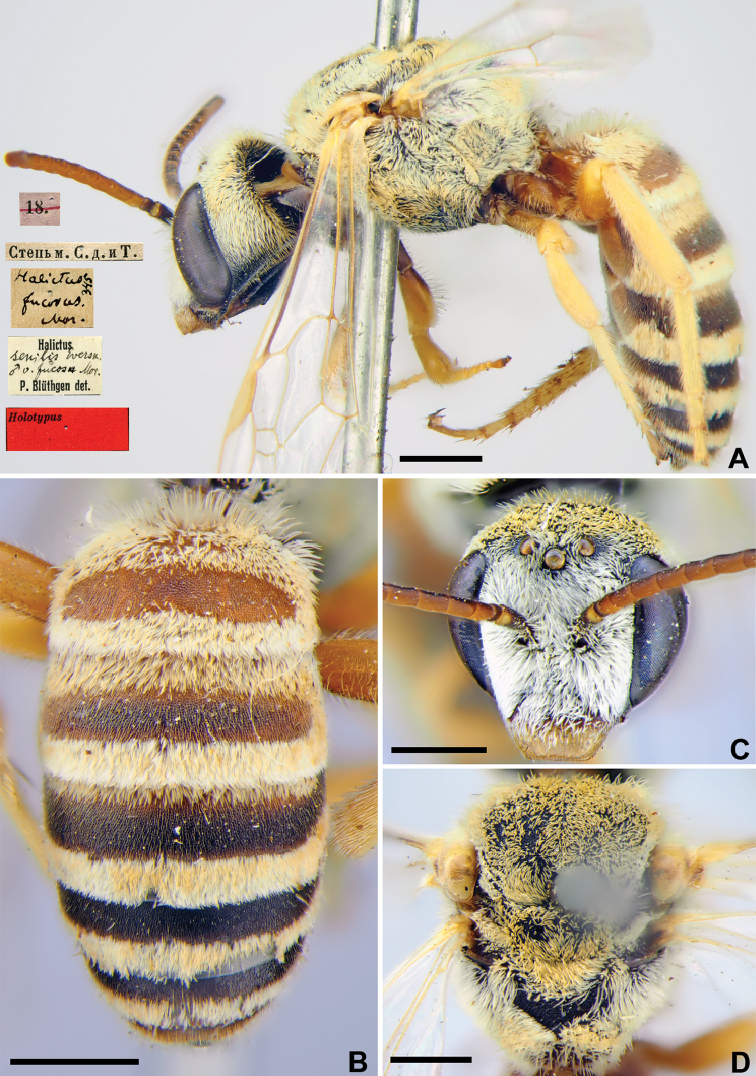
*Halictus fucosus* Morawitz, 1876, holotype, female **A** habitus, lateral view and labels **B** metasoma, dorsal view **C** head, frontal view **D** mesosoma, dorsal view. Scale bars: 1.0 mm.

##### 13. Halictus fulvitarsis

Taxon classificationAnimaliaHymenopteraHalictidae

Morawitz, 1876

D33FA76A-9B57-5EF7-8896-ED609504A6FA

Figure 12

Halictus fulvitarsis Morawitz, 1876: 219 (key), 239, ♂.

###### Type locality.

Khodzha-Chiburgan River (Tajikistan/Kyrgyzstan, not Uzbekistan as was indicated in Warncke 1982: 91).

###### Published (original) locality.

Tajikistan/Kyrgyzstan: Khodzha-Chiburgan River.

###### Holotype.

♂, 26.[VI.1871] // Чибурганъ [Tajikistan/Kyrgyzstan: Khodzha-Chiburgan River (near Vorukh, ≈ 39°48'N, 70°41'E) // *Halictus fulvitarsis* Mor., [N]361 [handwritten by F. Morawitz] // Holotypus <red label> [ZMMU].

###### Current status.

Lasioglossum (Lasioglossum) fulvitarse (Morawitz, 1876).

###### Remarks.

Description of male. Blüthgen 1934b: 147.

The lectotype designation by Warncke (1982: 91) is unnecessary as the species was described from a single male that was directly written about by Morawitz (1876: 240).

###### Distribution.

Tajikistan, Kyrgyzstan, Kazakhstan (Morawitz 1876, Pesenko 1986a, Murao et al. 2017).

**Figure 12.**
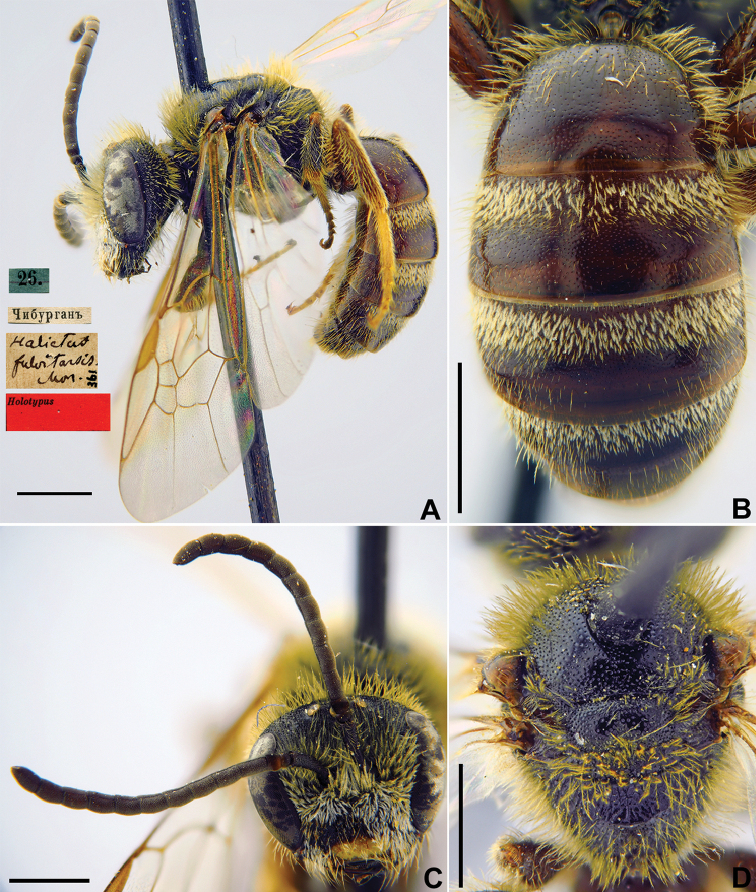
*Halictus fulvitarsis* Morawitz, 1876, holotype, female **A** habitus, lateral view and labels **B** metasoma, dorsal view **C** head, frontal view **D** mesosoma, dorsal view. Scale bars: 1.0 mm.

##### 14. Halictus funerarius

Taxon classificationAnimaliaHymenopteraHalictidae

Morawitz, 1876

BB3C364F-3185-508E-84E4-99096206C174

Figure (see Astafurova and Proshchalykin 2020: 414, figs 11a–e).

Halictus funerarius Morawitz, 1876: 217 (key), 235, ♀.

###### Type locality.

30 km SSE Samarkand, Sangu-dzhuman Pass (Uzbekistan).

###### Published (original) locality.

Uzbekistan: Sangu Dzhuman.

###### Lectotype.

♀, designated by Pesenko 1984: 21, <golden circle> // Сангы Джуманъ [Uzbekistan, Sangydzhuman Pass, 30 km SSE Samarkand, 39°22'N, 67°00'E] // 25.[V.1869] // *funerarius* Mor. Typ. [handwritten by F. Morawitz] // Lectotypus *H. funerarius* Mor., ♀, design. Pesenko [1]981 <red label> // Zoological Institute St. Petersburg INS_HYM_0000159 [ZISP].

###### Paralectotypes

(8 ♀). 8 ♀, the same label as in lectotype // Paralectotypus *H. funerarius* Mor. design. Pesenko [1]981 <red labels> [3 ♀ – ZISP; 5 ♀ – ZMMU].

###### Current status.

Halictus (Protohalictus) funerarius Morawitz, 1876.

###### Distribution.

A rare montane Central Asian species: Kazakhstan, Uzbekistan, Tajikistan, Western Kyrgyzstan, Iran, north-eastern Afghanistan and north-western China (Pesenko 2005a).

##### 15. Halictus fuscicollis

Taxon classificationAnimaliaHymenopteraHalictidae

Morawitz, 1876

871A8FE5-8CA9-5E23-9138-F676B77806EF

Figure 13

Halictus fuscicollis Morawitz, 1876: 217 (key), 229, ♀.

###### Type locality.

50 km NW Chardara, Kyzylkum Desert (Turkistan Province, Kazakhstan).

###### Published (original) locality.

Kazakhstan: “Kyzyl-Kum Steppe, near Baybek”.

###### Lectotype.

♀, designated by Warncke 1982: 137, 30.[IV.1871] // Кизилъкумъ [Kazakhstan, Baybek Well, Kyzylkum Desert, ca. 50 km NW Chardara, ≈ 41°44'N, 67°54'E] // *Halictus fuscicollis* Mor., [N]345 [handwritten by F. Morawitz] // *HalictusVestitohalictusfuscicollis* Mor., ♀, Lectotypus, design. A.W. Ebmer 1994 // Lectotypus *Halictus fuscicollis* Mor., design. Warncke <red label, labelled by Yu. Pesenko> [ZMMU].

###### Paralectotype.

1 ♀, <golden circle> Kisilkum [handwritten by F. Morawitz] // *fuscicollis* Mor. Typ. [handwritten by F. Morawitz] // Paralectotypus <red label>, labelled by Yu. Astafurova [ZISP].

###### Current status.

Halictus (Placidochalictus) fuscicollis Morawitz, 1876.

###### Remarks.

Description of male. Morawitz 1894: 68, as *Halictus flavocallosus* (synonymised by Blüthgen 1931a: 214).

###### Distribution.

Southern Kazakhstan, Turkmenistan, Iran, China (Xinjiang) (Morawitz 1876, 1894, Murao et al. 2017, Ascher and Pickering 2020).

**Figure 13.**
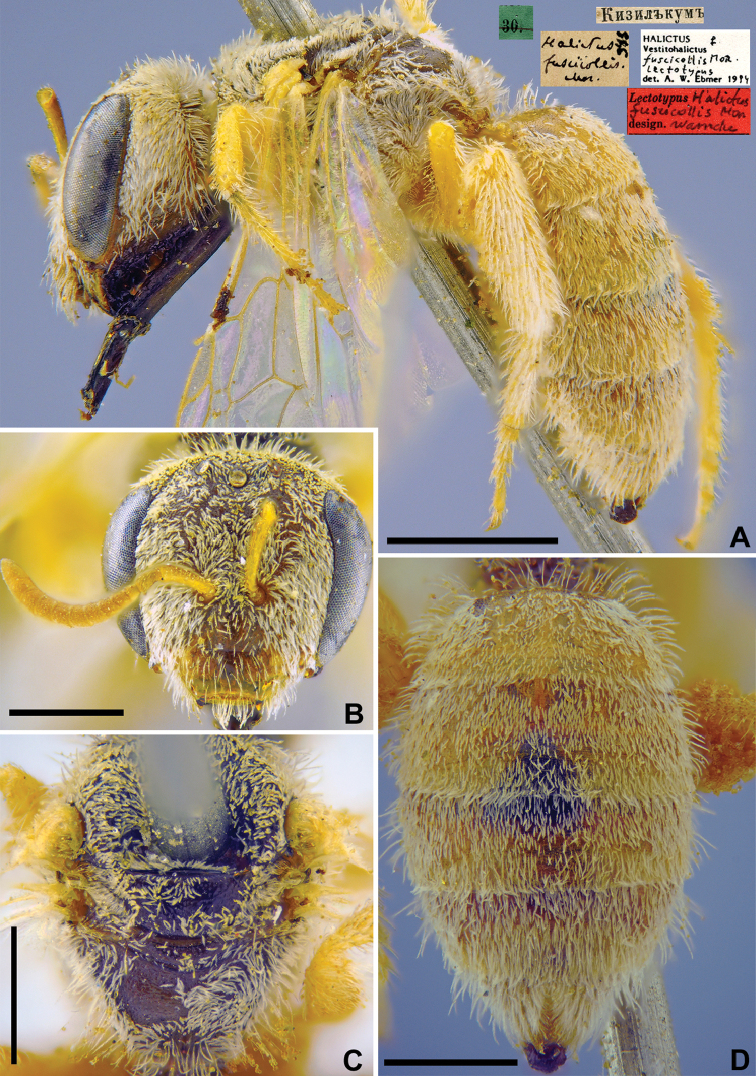
*Halictus fuscicollis* Morawitz, 1876, lectotype, female **A** habitus, lateral view and labels **B** head, frontal view **C** mesosoma, dorsal view **D** metasoma, dorsal view. Scale bars: 0.5 mm.

##### 16. Halictus hyalinipennis

Taxon classificationAnimaliaHymenopteraHalictidae

Morawitz, 1876

F8F496CE-73D6-5D1A-897C-ADA4523E76EF

Figure (see Astafurova and Proshchalykin 2018: 23, figs 21a–e).

Halictus hyalinipennis Morawitz, 1876: 218 (key to females), 220 (key to males), 253–254, ♀, ♂.

###### Type locality.

Tashkent (Uzbekistan).

###### Published (original) locality.

Kazakhstan: Chardara, steppe between Tashkent and Syr-Darya; Uzbekistan: Dzhamsk Gorge, Dzhizmansk Gorge, Ulus, Dzham, Urgut, Keles, Samarkand, Soch, Shakhimardan, Uch-Kurgan; Kyrgyzstan: Alay, Osh, Gulsha, Taka.

###### Lectotype.

♀, designated by Astafurova and Proshchalykin 2018: 22, Ташкентъ [Uzbekistan, Tashkent, 41°18'N 69°16'E] // *hyalinipennis* F. Mor., ♀ [handwritten by F. Morawitz] // к.[оллекция] Ф. Моравица [Collection of F. Morawitz] // Lectotypus *Halictus hyalinipennis* Morawitz, 1876, ♀, design. Astafurova & Proshchalykin 2018 <red label> [ZISP].

###### Paralectotypes

(29 ♀, 10 ♂). 1 ♀, Шагимарданъ [Shagimardan] // 3.[VII.1871] // к.[оллекция] Ф. Моравица [Collection of F. Morawitz]; 1 ♂, Така [Taka] // к.[оллекция] Ф. Моравица [Collection of F. Morawitz]; 1 ♀, 1 ♂, Сохъ [Sokh] // 29. [29.VI.1871] // к.[оллекция] Ф. Моравица [Collection of F. Morawitz]; 1 ♂, Уч-Курганъ [Uch- Kurgan] // к.[оллекция] Ф. Моравица [Collection of F. Morawitz]; 2 ♀, Ташкентъ [Tashkent] // к.[оллекция] Ф. Моравица [Collection of F. Morawitz] [ZISP]; 1 ♂, Ошъ [Osh] // 1.[VIII.1871]; 1 ♂, Самаркандъ [Samarkand] // 4.[VII.1869] // [N]384; 3 ♂, Самаркандъ [Samarkand] // 7.[VII.1869]; 2 ♀, Самаркандъ [Samarkand] // 3., 21.[III.1869]; 16 ♀, Ташкентъ [Tashkent] // 26., 27.[III.1871], 21., 23.[V.1871] and 1.,3.,5.,10.[VI.1871]; 1 ♀, Чардара [Chardara] // 27.[IV.1871]; 1 ♀, Учь-Курганъ [Uch-Kurgan] // 15.[VII.1871]; 2 ♂, Шагимарданъ [Shagimardan] // 2.[VII.1871] 5 ♀, Заравшан.[ская] дол.[ина] [Zeravshan River valley], 3., 11., 18., 23.[III.1871] // Paralectotypus *Halictus hyalinipennis* Morawitz, 1876, design. Astafurova & Proshchalykin 2018 <identical red labels on each paralectotype specimen> [ZMMU].

###### Current status.

Lasioglossum (Sphecodogastra) hyalinipenne (Morawitz, 1876).

###### Distribution.

Iran, Afghanistan, Kazakhstan, Uzbekistan, Kyrgyzstan, Tajikistan (Morawitz 1876, Ebmer 1974, Warncke 1982, Murao et al. 2017).

##### 17. Halictus laevinodis

Taxon classificationAnimaliaHymenopteraHalictidae

Morawitz, 1876

7C938E0B-F404-5BEB-BC69-F48764E583B4

Figure (see Astafurova and Proshchalykin 2018: 24, figs 23a–e).

Halictus laevinodis Morawitz, 1876: 218 (key to females), 248, ♀.

###### Type locality.

30 km SSE Samarkand, Sangu-dzhuman Pass (Uzbekistan).

###### Published (original) locality.

Uzbekistan: Sangy Dzhuman.

###### Lectotype (designated here).

♀, <golden circle> // Сангы Джуманъ [Uzbekistan, Sangy-dzhuman Pass, 30 km SSE Samarkand, Zeravshan Ridge, 39°27'N, 67°14'E] // 25.[III.1869] // *laevinodis* Mor., Typ. [handwritten by F. Morawitz] // Lectotype *Halictus laevinodis*, design. Astafurova et Proshchalykin, 2020 <red label> [ZISP].

###### Paralectotypes

(2 ♀). 1 ♀, 25.[III.1869] // Сангы Джуманъ [Sangy Dzhuman] // *Halictus laevinodis* Mor. [handwritten by F. Morawitz]; 1 ♀ 25.[III.1869] // Сангы Джуманъ [Sangy Dzhuman] // [N]375 // Paralectotype *Halictus laevinodis*, design. Astafurova et Proshchalykin, 2020 <identical red labels on each paralectotype specimen> [ZMMU].

###### Current status.

Lasioglossum (Hemihalictus) laevinode (Morawitz, 1876).

###### Remarks.

Astafurova and Proshchalykin (2018: 23) indicated the lectotype specimen as «Holotype», but Morawitz (1876) did not directly indicate a single specimen and two specimens from the type series are deposited in ZMMU.

Description of male. Blüthgen, 1934b: 154, Fig. 3.

###### Distribution.

Kazahkstan, Uzbekistan, Tajikistan, Kyrghyzstan, Afghanistan (Morawitz 1876, Blüthgen in Popov 1935, Ebmer 1980, Murao et al. 2017).

##### 18. Halictus limbellus

Taxon classificationAnimaliaHymenopteraHalictidae

Morawitz, 1876

0CB23044-6369-5D2D-A43E-C7004EE64026

Figure 14

Halictus limbellus Morawitz, 1876: 218 (key), 249, ♀.

###### Type locality.

Samarkand (Uzbekistan).

###### Published (original) locality.

Uzbekistan: Samarkand; Tajikistan: Peti.

###### Lectotype (designated here).

♀, 5.[IV.1869] // Самаркандъ [Uzbekistan, Samarkand, 39°39'N, 66°57'E] // *Halictus limbellus* Mor., [N]377 [handwritten by F. Morawitz] // Lectotypus *Halictus limbellus* Mor., Astafurova et Proshchalykin, 2020 <red label> [ZMMU].

###### Paralectotypes

(3 ♀). 1 ♀, 5.[IV.1869] // Самаркандъ [Samarkand] // [N]377 // Typus <red label> [ZMMU]; 1 ♀, <golden circle> // 5.[IV.1869] // Самаркандъ [Samarkand] // *Halictus limbellus* F. Mor., Typ. [handwritten by F. Morawitz]; 1 ♀, Самаркандъ [Samarkand] // к.[оллекция] Ф. Моравица [Collection of F. Morawitz] // *limbellus* Mor., Typ. [handwritten by F. Morawitz] // Paralectotypus *Halictus limbellus* Mor., Astafurova et Proshchalykin, 2020 <identical red labels on each paralectotype specimen> [ZISP].

###### Current status.

Lasioglossum (Hemihalictus) limbellum (Morawitz, 1876).

###### Remarks.

Description of male. Blüthgen 1930: 763.

The lectotype designation by Warncke (1982: 69) is invalid because he labelled no female of the two females from Samarkand deposited in ZMMU.

###### Distribution.

Central and eastern Europe, Turkey, Caucasus, Russia (North Caucasus), Israel, Iran, Afghanistan, Tajikistan, Uzbekistan, Kazakhstan, China (Gansu) (Pesenko 2007, Astafurova and Proshchalykin 2017, Murao et al. 2017).

**Figure 14.**
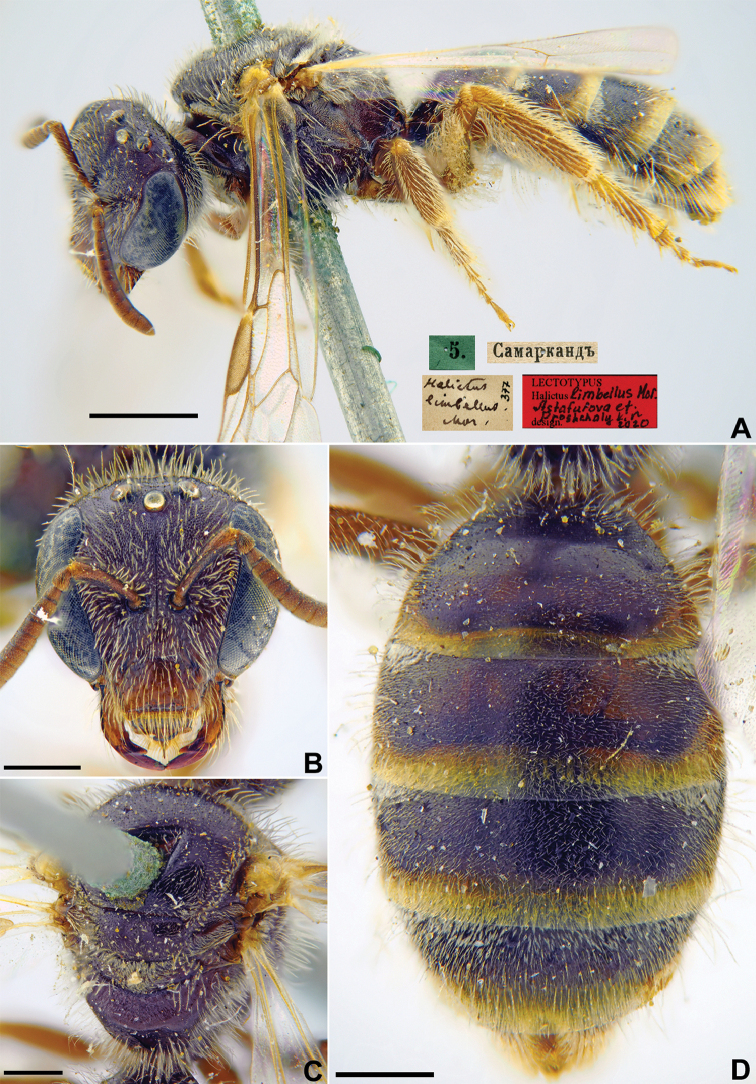
*Halictus limbellus* Morawitz, 1876, lectotype, female **A** habitus, lateral view and labels **B** head, frontal view **C** mesosoma, dorsal view **D** metasoma, dorsal view. Scale bars: 1.0 mm (**A**), 0.5 mm (**B–D**).

##### 19. Halictus longirostris

Taxon classificationAnimaliaHymenopteraHalictidae

Morawitz, 1876

FA1223E9-2585-5D0A-A360-4B8E58FC0ADC

Figure 15

Halictus longirostris Morawitz, 1876: 216 (key to females), 219 (key to males), 236, ♀, ♂.

###### Type locality.

Shakhimardan (Uzbekistan).

###### Published (original) locality.

Uzbekistan: on the road to Sangy-dzhuman Pass, Shakhimardan; Tajikistan: Peti.

###### Lectotype.

♂, designated by Warncke 1982: 80, 3.[VII.1871] // Шагимарданъ [Shakhimardan in the Uzbek enclave in the territory of Kyrgyzstan, Alai Ridge; 39°58'N, 71°47'E] // *Halictus longirostris* Mor., [N]356 [handwritten by F. Morawitz] // Lectotypus *Halictus longirostris* Mor., design. Warncke [19]82 <red label, labelled by Yu. Astafurova> [ZMMU].

###### Paralectotypes

(2 ♀). 1 ♀, 25.[V.1869] // Сангы Джуманъ [Sangy Dzhuman] // [N]356; 1 ♀, 12.[VII.1870] // Фанъ [Fan] // [N]356 [ZMMU]; 1 ♀, 2.[VII.1871] // Шагимарданъ [Shagimardan] // к.[оллекция] Ф. Моравица [Collection of F. Morawitz] // *Halictus longirostris* Mor. [handwritten by F. Morawitz] // Paralectotypus *Halictus longirostris* Mor., design. Warncke < identical red labels on each paralectotype specimen, labelled by Yu. Astafurova> [ZISP].

###### Current status.

Lasioglossum (Hemihalictus) longirostre (Morawitz, 1876).

###### Distribution.

Greece, Israel, Lebanon, Turkey, Caucasus, Iran, Afghanistan, Central Asia, Kazakhstan, China (Xinjiang) (Murao et al. 2017, Niu et al. 2020).

**Figure 15.**
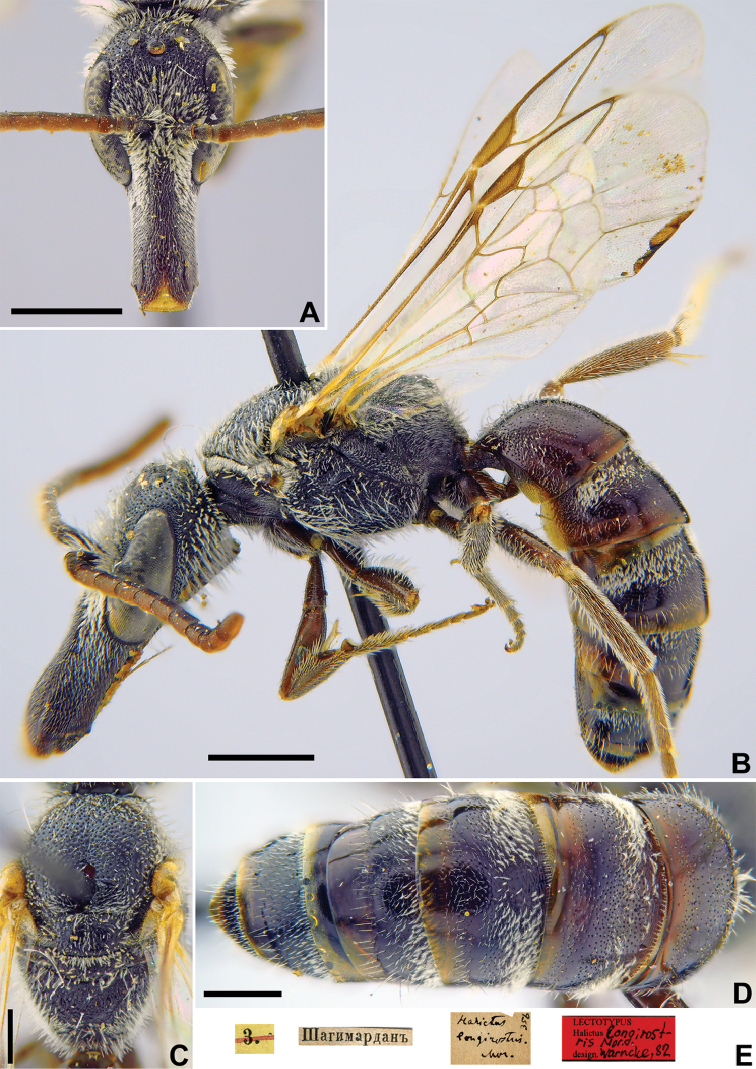
*Halictus longirostris* Morawitz, 1876, lectotype, male **A** head, frontal view **B** habitus, lateral view **C** mesosoma, dorsal view **D** metasoma, dorsal view **E** labels. Scale bars: 1.0 mm (**A, B**), 0.5 mm (**C, D**).

##### 20. Halictus maculipes

Taxon classificationAnimaliaHymenopteraHalictidae

Morawitz, 1876

B576028E-08FD-52B4-A80E-8B044BCC73C2

Figure 16

Halictus maculipes Morawitz, 1876: 218 (key to females), 247, ♀.

###### Type locality.

Sokh District [Uzbekistan].

###### Published (original) locality.

Kekh [Sokh District, Uzbekistan].

###### Lectotype.

♀ designated by Warncke 1982: 68, <golden circle> // 27.[VI.1871] // Сохъ [Uzbekistan, Sokh District, ≈ 39°57'N, 71°07'E] // *Halictus maculipes* Mor., [N]373 [handwritten by F. Morawitz] // Lectotypus *Halictus maculipes* Mor., design. Warncke <red label, labelled by Yu. Astafurova> [ZMMU].

###### Current status.

Lasioglossum (Hemihalictus) maculipes (Morawitz, 1876).

###### Remarks.

Male unknown.

###### Distribution.

Turkey, Turkmenistan, Tajikistan, Uzbekistan, Iran, Afghanistan (Morawitz 1876, Ebmer 1984, 1986, Warncke 1982, Ascher and Pickering 2020).

**Figure 16.**
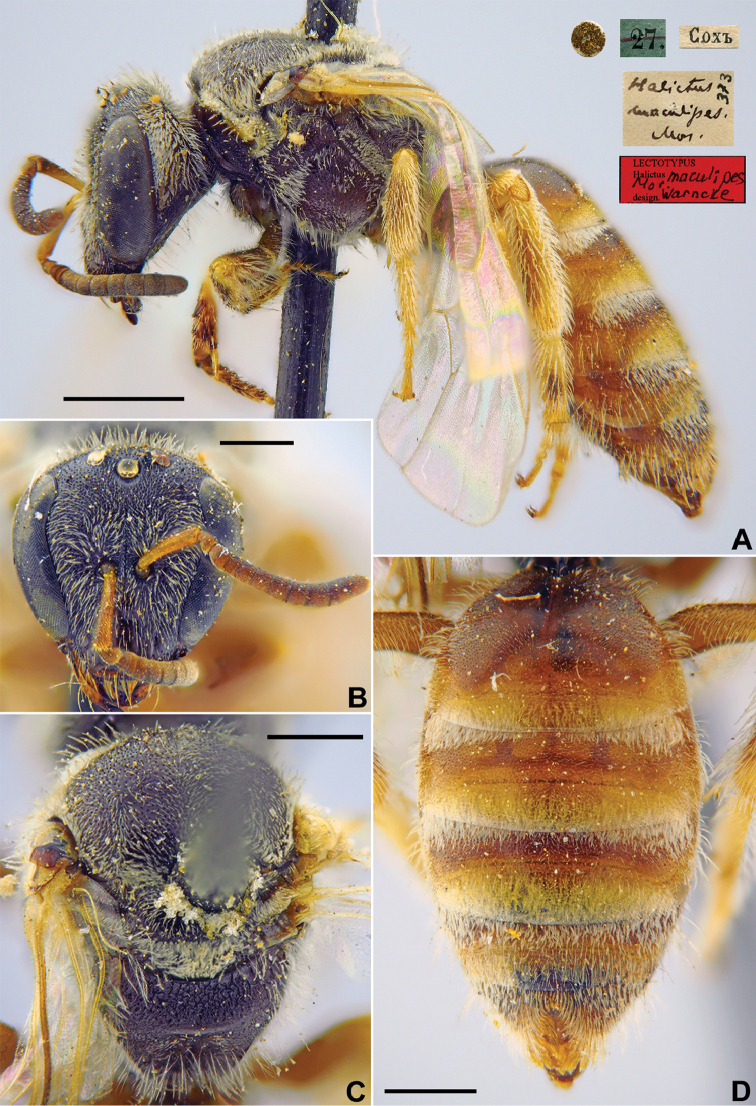
*Halictus maculipes* Morawitz, 1876, lectotype, female **A** habitus, lateral view and labels **B** head, frontal view **C** mesosoma, dorsal view **D** metasoma, dorsal view. Scale bars: 1.0 mm (**A**), 0.5 mm (**B–D**).

##### 21. Halictus melanarius

Taxon classificationAnimaliaHymenopteraHalictidae

Morawitz, 1876

13F40084-10DA-56C1-A4F2-92B7254C56F6

Figure 17

Halictus melanarius Morawitz, 1876: 241, ♂.

###### Type locality.

Shakhimardan (Uzbekistan).

###### Published (original) locality.

Near Shakhimardan.

###### Holotype.

♂, 9.[VII.1871] // Шагимарданъ [Shakhimardan in the Uzbek enclave in the territory of Kyrgyzstan, Alai Ridge; 39°58'N, 71°47'E] // *Halictus melanarius* Mor., [N]364 [handwritten by F. Morawitz] // *Lasioglossum fallax* (Mor.) syn: *melanarium* (Mor.) det. A.W. Ebmer 1979 // Holotypus <red label> [ZMMU].

###### Current status.

Lasioglossum (Lasioglossum) fallax ssp. melanarium (Morawitz, 1876) (subspecies status according to Ebmer 1998: 382).

###### Remarks.

Description of female: Ebmer 1980: 493, Figs 12 and 13, as *Lasioglossum melan* Ebmer, 1980 (synonymised by Ebmer 1998: 382).

The lectotype designations by Ebmer (1980: 495) and by Warncke (1982: 91) were unnecessary as the species was described from a single male that was directly written about by Morawitz (1876: 241).

###### Distribution.

Kazakhstan, Uzbekistan, Tajikistan, Kyrgyzstan, Afghanistan, Mongolia (Hovd) (Pesenko 2006b).

**Figure 17.**
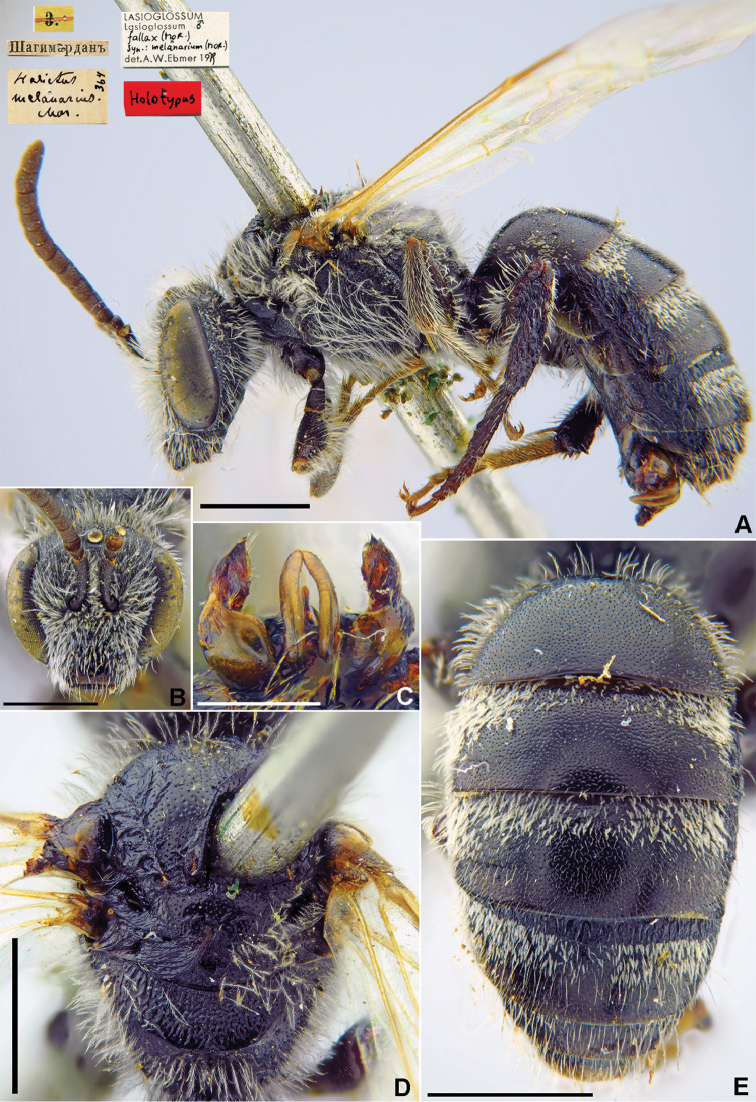
*Halictus melanarius* Morawitz, 1876, holotype, male **A** view habitus, lateral view and labels **B** head, frontal **C** genitalia, dorsal view **D** mesosoma, dorsal view **E** metasoma, dorsal view. Scale bars: 1.0 mm (**A, B, D, E**), 0.5 mm (**C**).

##### 22. Halictus minor

Taxon classificationAnimaliaHymenopteraHalictidae

Morawitz, 1876

B9D20F93-7D49-5662-B92C-95BD9EA40A1A

Figure (see Astafurova and Proshchalykin 2020: 418, figs 15a–e).

Halictus minor Morawitz, 1876: 217 (key), 233, ♀.

###### Type locality.

30 km SSE Samarkand, Sangu-dzhuman Pass (Uzbekistan).

###### Published (original) locality.

Uzbekistan: Gus [near Urgut], Sangy-Dzhuman; Tajikistan: Pyandzhikent.

###### Lectotype.

♀, designated by Pesenko 1984: 23, <golden circle> // Сангы Джуманъ [Uzbekistan, Sangy-dzhuman Pass, 30 km SSE Samarkand, 39°20'N, 67°19'E] // 25.[V.1869] // *minor* Mor. [handwritten by F. Morawitz] // Lectotypus *H. minor* Mor., design. Pesenko, [1]981, ♀ <red label> // Zoological Institute St. Petersburg INS_HYM_0000164 [ZISP].

###### Paralectotypes

(3 ♀). 1 ♀, Сангы Джуманъ [Sangy Dzhuman] // к.[оллекция] Ф. Моравица [Collection of F. Morawitz] // *minor* Mor. [handwritten by F. Morawitz] // Syn.: *jarkandensis* Strand, ♀ // Paralectotypus *H. minor* Mor., design. Pesenko, [1]981, ♀ <red label >[ZISP]; 1 ♀, 25.[V.1869] // Сангы Джуманъ [Sangy Dzhuman] // *Halictus minor* Mor., [N]352 [handwritten by F. Morawitz]; 1 ♀, 24.[V.1869] // Заравшан.[ская] дол.[ина] [Zeravshan River valley, Gus] // [N]352 // Paralectotypus *H. minor* Mor., design. Pesenko, [1]981, ♀ <identical red labels on each paralectotype specimen> [ZMMU].

###### Current status.

Halictus (Platyhalictus) minor Morawitz, 1876.

###### Remarks.

Description of male. Blüthgen 1936: 295, fig. 11.

###### Distribution.

Azerbaijan, Afghanistan, Iran, Kazakhstan, Central Asia, Altai, Pakistan, north-western and northern China, northern India (Pesenko 2005a, b).

##### 23. Halictus modernus

Taxon classificationAnimaliaHymenopteraHalictidae

Morawitz, 1876

2CE15736-D68B-502E-9EAE-6318C69E3B1F

Figure 18

Halictus modernus Morawitz, 1876: 217 (key), 235, ♀.

###### Type locality.

Samarkand (Uzbekistan).

###### Published (original) locality.

Uzbekistan: near Samarkand.

###### Holotype.

♀, 5.[VII.1870] // Самаркандъ [Uzbekistan, Samarkand, 39°39'N, 66°57'E] // *Halictus modernus* Mor., [N]354 [handwritten by F. Morawitz] // Holotypus <red label> [ZMMU].

###### Current status.

Halictus (Lampralictus) modernus Morawitz, 1876.

###### Remarks.

The lectotype designation of Warncke (1982: 147) is unnecessary as the species was described from a single female that was directly written about by Morawitz (1876: 235).

Description of male. Ebmer 1984: 315, figs 3–5.

###### Distribution.

Turkmenistan, Uzbekistan, Kyrgyzstan, Afghanistan, Pakistan (Pesenko 2005a).

**Figure 18.**
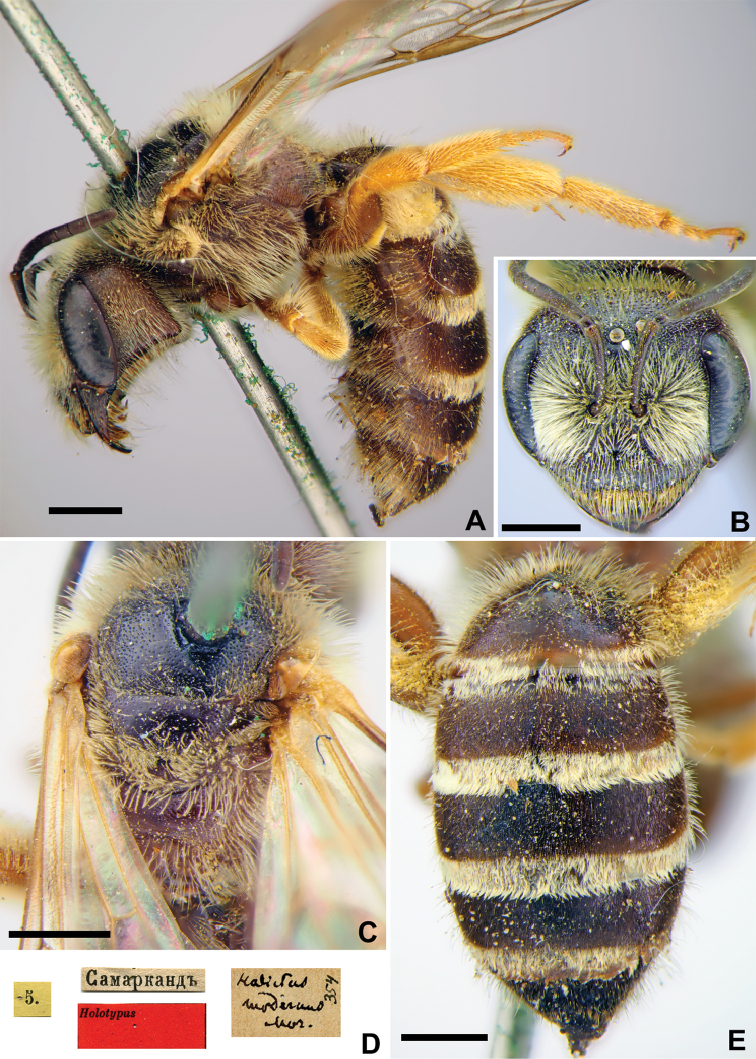
*Halictus modernus* Morawitz, 1876, holotype, female **A** habitus, lateral view **B** head, frontal view **C** mesosoma, dorsal view **D** labels **E** metasoma, dorsal view. Scale bars: 1.0 mm.

##### 24. Halictus nasica

Taxon classificationAnimaliaHymenopteraHalictidae

Morawitz, 1876

D85BC9E9-13A7-5DF7-BC2E-5BDE6246F0E3

Figure 19

Halictus nasica Morawitz, 1876: 216 (key to females), 219 (key to males), 229, ♀, ♂.

###### Type locality.

Samarkand (Uzbekistan).

###### Published (original) locality.

Southern Kazakhstan: Kysyl-Kum [desert] near draw-well Chakany; Uzbekistan: steppe between Katty-Kurgan and Ulus, Samarkand, Murzarabat, Chinaz, Sokh.

###### Lectotype (designated here).

♀, 9.[VI.1869] // Заравшан.[ская] дол.[ина] [Uzbekistan, Zeravshan River valley, near Samarkand, 39°39'N, 66°57'E] // *Halictus nasica* Mor., [N]346 [handwritten by F. Morawitz] // Lectotypus *Halictus nasica* Mor., design. Astafurova et Proshchalykin, 2020 <red label> [ZMMU].

###### Paralectotypes

(28 ♀, 44 ♂). 14 ♀, the same label as in the lectotype; 11 ♀, 2 ♂, 9., 13.[VI.1869], 4., 7.[VII.1869] // Самаркандъ [Samarkand] // [N]346; 12 ♂, 24.[VII.1870], 29.[VIII.1870] // Мурзарабадъ [Murzarabad] // [N]346; 1 ♀, 9 ♂, 28.[IV.1871] // Кизилъкумъ [Kizilkum] //[N]346; 17 ♂, 25.[VII.1870] // Чиназъ [Chinaz] // [N]346 [ZMMU]; 1 ♀, 28.[IV.1871] // Кизилъкумъ [Kizilkum] // к.[оллекция] Ф. Моравица [Collection of F. Morawitz] // *Halictus nasica* Mor. [handwritten by F. Morawitz]; 1 ♀, <golden circle> // 9.[VI.1869] // Заравшан.[ская] дол.[ина] [Zeravshan River valley, near Samarkand] // *nasica* Mor. Typ. [handwritten by F. Morawitz]; 3 ♂, 28.[VI.1871] // Сохъ [Sokh] // к.[оллекция] Ф. Моравица [Collection of F. Morawitz]; 1 ♂, 9.[VI.1869] // Самаркандъ [Samarkand] // к.[оллекция] Ф. Моравица [Collection of F. Morawitz] // Paralectotypus *Halictus nasica* Mor., design. Astafurova et Proshchalykin, 2020 <identical red labels on each paralectotype specimen> [ZISP].

###### Current status.

Halictus (Vestitohalictus) nasica Morawitz, 1876.

###### Remarks.

The lectotype designation by Warncke (1982: 138) is invalid because he labelled none of the 15 females from “valle Serafshan” deposited in ZMMU.

###### Distribution.

Morocco, Kazakhstan, Uzbekistan, Turkmenistan, Iran, Afghanistan, Pakistan (Murao et al. 2017, Ascher and Pickering 2020).

**Figure 19.**
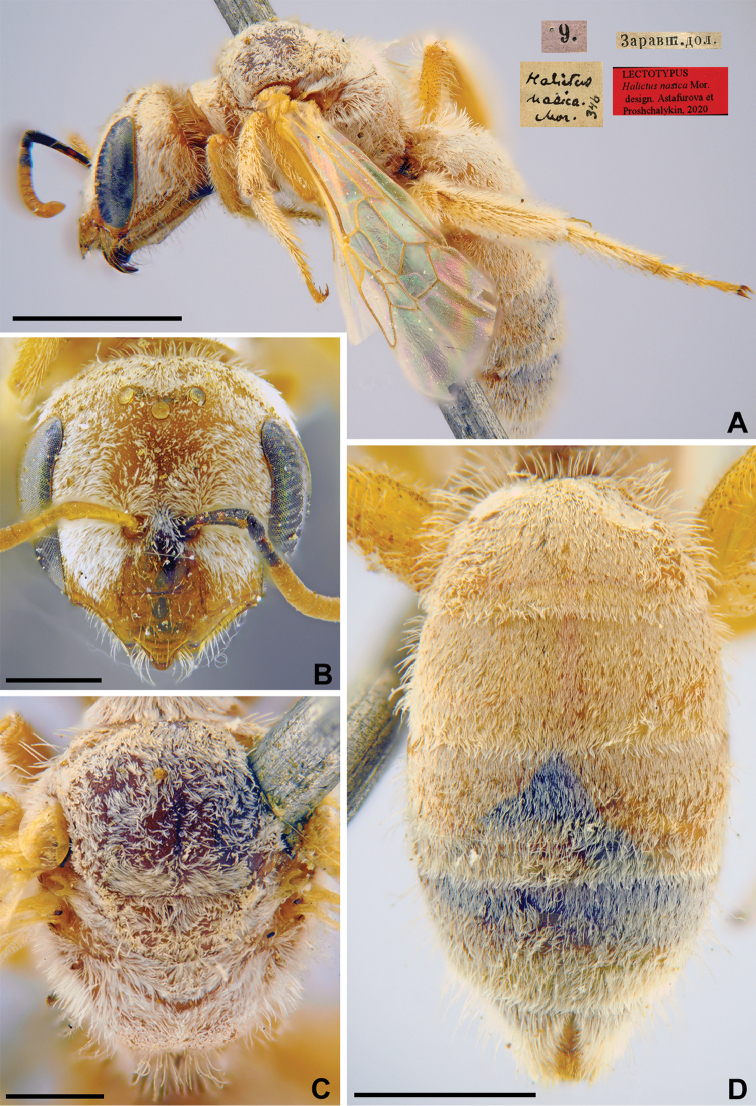
*Halictus nasica* Morawitz, 1876, lectotype, female **A** habitus, lateral view and labels **B** head, frontal view **C** mesosoma, dorsal view **D** metasoma, dorsal view. Scale bars: 1.0 mm (**A, D**), 0.5 mm (**B, C**).

##### 25. Halictus nigrilabris

Taxon classificationAnimaliaHymenopteraHalictidae

Morawitz, 1876

6F363A18-5778-5D9F-A346-BA56996CDC92

Figure 20

Halictus nigrilabris Morawitz, 1876: 249, ♂.

###### Type locality.

Sarafschan River valley, Yeri (Tajikistan).

###### Published (original) locality.

Tajikistan: valley Sarafschan between Iori and Dashty-Kazy.

###### Lectotype.

♂, designated by Warncke 1982: 91, <golden circle> // 31.[V.1869] // Заравшан.[ская] дол.[ина] [Tajikistan, Zeravshan River valley, near Iori (= Yeri), 39°29'N, 67°53'E] // *Halictus nigrilabris* Mor., [N]378 [handwritten by F. Morawitz] // Lectotypus *Halictus nigrilabris* Mor. design. Warncke [1]982 <red label, labelled by Yu. Pesenko> [ZMMU].

###### Paralectotype.

1 ♂, Заравшан.[ская] дол.[ина] [Zeravshan River valley] // *Halictus nigrilabris* F. Morawitz [handwritten by F. Morawitz] // к.[оллекция] Ф. Моравица [Collection of F. Morawitz] // Paralectotypus *Halictus nigrilabris* Mor., design. Warncke [1]982 <red label, labelled by Yu. Pesenko> // Zoological Institute St. Petersburg, INS_HYM 0000076 [ZISP].

###### Current status.

Lasioglossum (Lasioglossum) nigrilabre (Morawitz, 1876).

###### Remarks.

Description of female. Blüthgen 1931b: 336, as *Halictus subprasinus* (synonymised by Ebmer 1978: 39).

###### Distribution.

Iran, Afghanistan, Turkmenistan, Uzbekistan, Tajikistan, Kyrgyzstan (Blüthgen 1931b, Pesenko 1986a).

**Figure 20.**
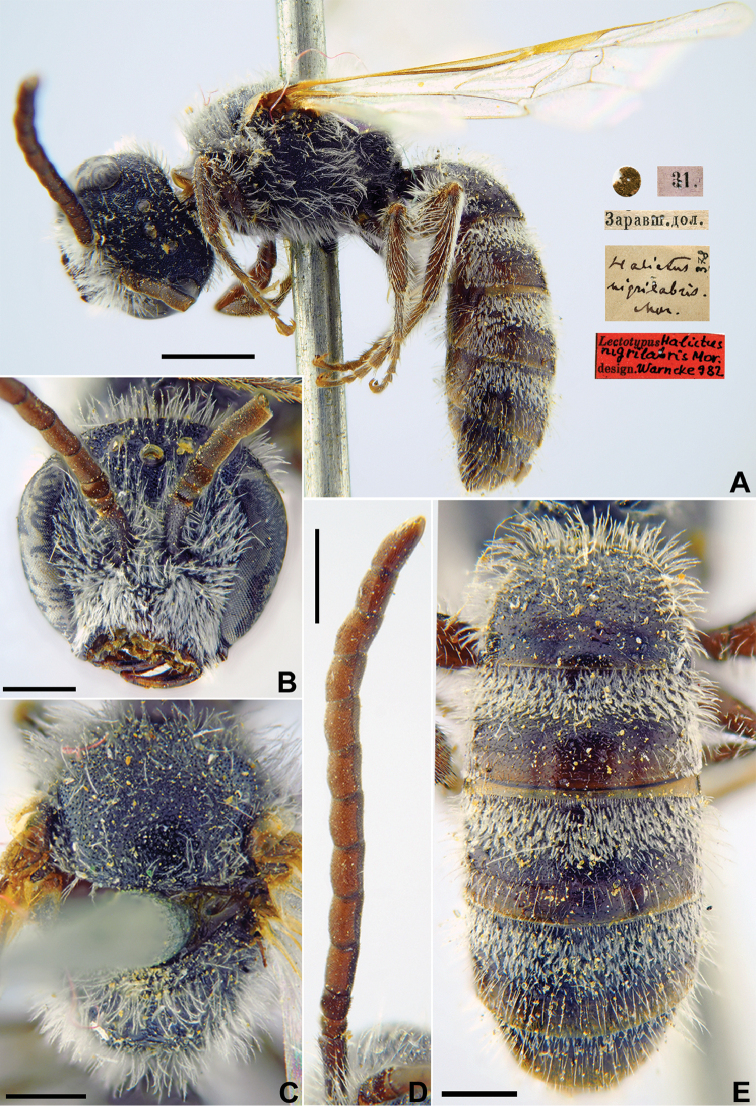
*Halictus nigrilabris* Morawitz, 1876, lectotype, male **A** habitus, lateral view and labels **B** head, frontal view **C** mesosoma, dorsal view **D** antennae, dorsal view **E** metasoma, dorsal view. Scale bars: 1.0 mm.

##### 26. Halictus nigripes

Taxon classificationAnimaliaHymenopteraHalictidae

Morawitz, 1876

24CFCD7C-6EAB-522D-8E03-B15E80836F2A

Figure 21

Halictus nigripes Morawitz, 1876: 251, ♂.

###### Type locality.

Karazuk, vicinity of Shakhimardan (Uzbekistan).

###### Published (original) locality.

Tajikistan: Iori George, Iskander River; Uzbek enclave in Kyrgyzstan: Karakazuk; Kyrgyzstan: Alay.

###### Lectotype.

♂, designated by Blüthgen 1934a: 302, 11.[VII.1871] // Каразукъ [Uzbekistan, Karazuk, vicinity of Shakhimardan 39°60'N, 71°50'E] // *Halictus nigripes* Mor., [N]380 [handwritten by F. Morawitz] // *nigripes* Mor., ♂, lecto-holotype, Blüthgen det. 1933 // Lectotypus *Halictus nigripes* Mor., design. Blüthgen [19]34 <red label> labelled by Yu. Astafurova [ZMMU].

###### Paralectotypes

(3 ♂). 1 ♂, 23.[VII.1871] // Алай [Alay] // [N]380 // *nigripes* Mor. ♂, Lecto-Paratype, Blüthgen det., 1933 [ZMMU]; 1 ♂, <golden circle> // 23.[VII.1871] // Алай [Alay] // *nigripes* Mor. Typ., [N]380 [handwritten by F. Morawitz]; 1 ♂, 22.[VII.1871] // Алай [Alay] // к.[оллекция] Ф. Моравица [Collection of F. Morawitz] // *Halictus nigripes* Mor. [handwritten by F. Morawitz] // Paralectotypus *Halictus nigripes* Mor., design. Blüthgen <identical red labels on each paralectotype specimen, labelled by Yu. Astafurova> [ZISP].

###### Current status.

Lasioglossum (Hemihalictus) melanopus (Dalla Torre, 1896), replacement name for *Halictus nigripes* Morawitz, 1876 (nec *H. nigripes* Lepeletier, 1841).

###### Remarks.

The specimens from Iskander and Iori George in the Morawitz type series are the holotype and paratypes of *Halictus pseudonigripes* Blüthgen, 1934.

Description of female: Vachal 1902: 229, as *Halictus attritus* (synonymised by Blüthgen 1934a: 301).

###### Distribution.

Uzbekistan, Kyrgyzstan, Kazakhstan, Tajikistan, Afghanistan, China (Xinjiang) (Morawitz 1876, Murao et al. 2017, Ascher and Pickering 2020).

**Figure 21.**
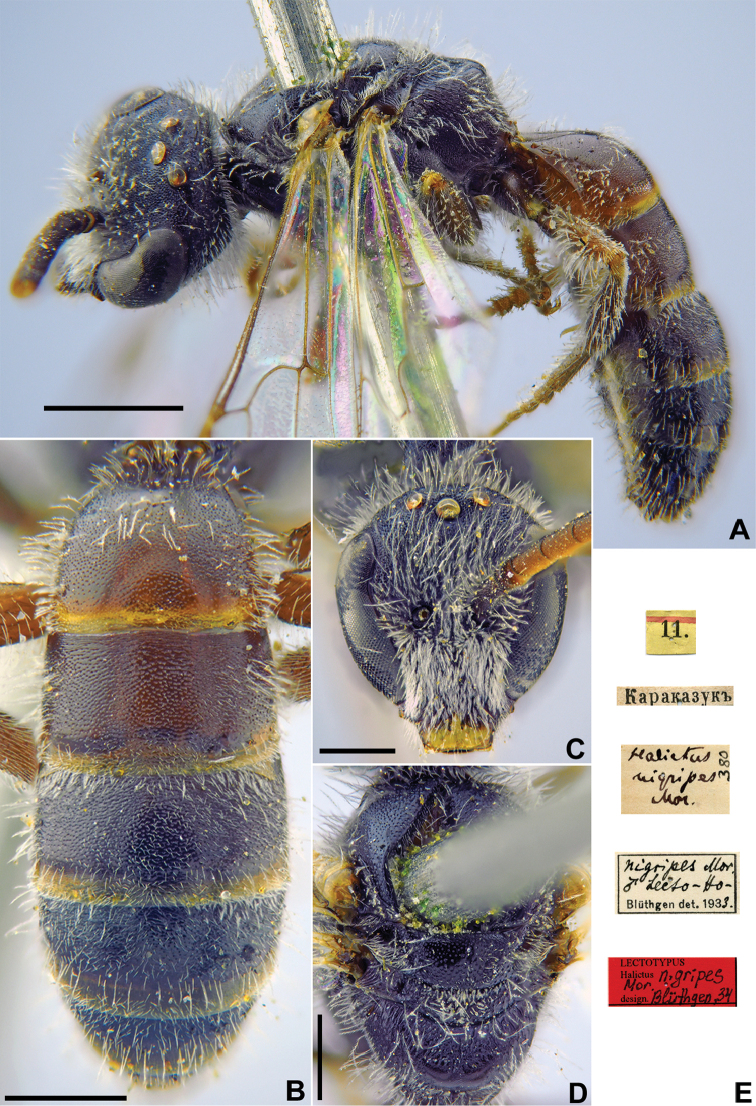
*Halictus nigripes* Morawitz, 1876, lectotype, male **A** habitus, lateral view **B** metasoma, dorsal view **C** head, frontal view **D** mesosoma, dorsal view **E** labels. Scale bars: 1.0 mm (**A**), 0.5 mm (**B–D**).

##### 27. Halictus obscuratus

Taxon classificationAnimaliaHymenopteraHalictidae

Morawitz, 1876

EBA6D4CC-95C9-543D-957F-98FFF03A1131

Figure 22

Halictus obscuratus Morawitz, 1876: 218 (key), 245, ♀.

###### Type locality.

Sangy-dzhuman Pass, 30 km SSE Samarkand [Uzbekistan].

###### Published (original) locality.

Uzbekistan: Samarkand, Aksay; Tajikistan: Iori Gorge, Varzaminor [=Ayni], Sangy-Dzhuman Pass.

###### Lectotype.

♀, designated by Warncke 1982: 116, 25.[V.1869] // Сангы Джуманъ [Uzbekistan, Sangy-dzhuman Pass, 30 km SSE Samarkand, 39°20'N, 67°19'E] // *Halictus obscuratus* Mor., [N]370 [handwritten by F. Morawitz] // Lectotypus *Halictus obscuratus* Mor., design. Warncke [19]82 <red label, labelled by Yu. Astafurova> [ZMMU].

###### Paralectotypes

(7 ♀). 1 ♀, 3.[IV.1869] // Самаркандъ [Samarkand] // [N]370; 1 ♀, 27.[II.1869] // Самаркандъ [Samarkand] // [N]370 // *obscuratus* [handwritten by F. Morawitz]; 1 ♀, [7.VI.1870] // Варзаминоръ [Varzaminor] // [N]370; 1 ♀, 16.[V.1869] // Заравшан.[ская] дол.[ина] [Zeravshan River valley, Aksay] // N[370]; 1 ♀, 2.[VI.1869] // Заравшан.[ская] дол.[ина] [Zeravshan River valley, Iori Gorge] // N[370]; [ZMMU]; 1 ♀, <golden circle> // 25.[V.1869] // Сангы Джуманъ [Sangy Dzhuman] // *obscuratus* Mor., Typ. [handwritten by F. Morawitz]; 1 ♀, Сангы Джуманъ [Sangy Dzhuman] // к.[оллекция] Ф. Моравица [Collection of F. Morawitz] // *obscuratus* Mor.[handwritten by F. Morawitz]; 1 ♀, Заравшан.[ская] дол.[ина] [Zeravshan River valley] // к.[оллекция] Ф. Моравица [Collection of F. Morawitz] // *Halictus obscuratus* F. Morawitz, ♀ [handwritten by F. Morawitz] // Paralectotypus *Halictus obscuratus* Mor., design. Warncke <identical red labels on each paralectotype specimen, labelled by Yu. Astafurova> [ZISP].

###### Current status.

Lasioglossum (Sphecodogastra) obscuratum ssp. obscuratum (Morawitz, 1876).

###### Remarks.

Description of male. Blüthgen 1923a: 277.

###### Distribution.

Europe (except North), Cyprus, Azerbaijan, Russia (North Caucasus), Turkey, Syria, Jordan, Israel, Iran, Afghanistan, Central Asia, Kazakhstan (Astafurova and Proshchalykin 2017).

**Figure 22.**
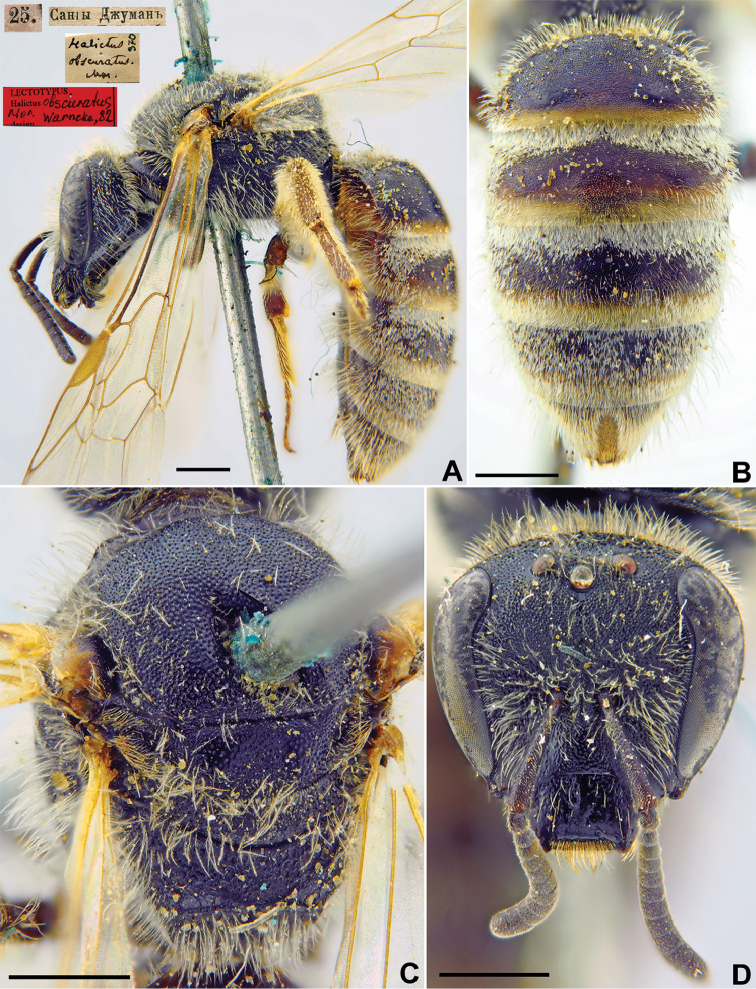
*Halictus obscuratus* Morawitz, 1876, lectotype, female **A** habitus, lateral view and labels **B** metasoma, dorsal view **C** mesosoma, dorsal view **D** head, frontal view **E**. Scale bars: 1.0 mm.

##### 28. Halictus palustris

Taxon classificationAnimaliaHymenopteraHalictidae

Morawitz, 1876

F01DBEB8-81F4-50B8-A2AE-D44EEF813413

Figure 23

Halictus palustris Morawitz, 1876: 217 (key), 234, ♀.

###### Type locality.

Iskanderkul Lake (Tajikistan).

###### Published (original) locality.

Tajikistan: “near Iskander-Kul Lake”.

###### Lectotype.

♀, designated by Warncke 1982: 147, <golden circle> // 15.[VI.1870] // Искандеръ [Tajikistan, Iskanderkul Lake, Hissar Ridge, 39°04'N, 68°22'E] // *Halictus palustris* Mor., [N]353 [handwritten by F. Morawitz] // Lectotypus *Halictus palustris* Mor., ♀, design. Warncke [1]982 <red label>, labelled by Yu. Pesenko [ZMMU].

###### Paralectotype.

1 ♀, <golden circle> // 15.[VI.1870] // Искандеръ [Iskander] // *palustris* Mor., Typ. [handwritten by F. Morawitz] // Paralectotypus *H. palustris* Mor., ♀, design. Pesenko [1]981 <red label> [ZISP].

###### Current status.

Halictus (Tytthalictus) palustris Morawitz, 1876.

###### Remarks.

Description of male. Blüthgen 1936: 291.

###### Distribution.

Kazakhstan, Uzbekistan, Tajikistan, Kyrgyzstan, China (Xinjiang) (Pesenko 1986b, Murao et al. 2017).

**Figure 23.**
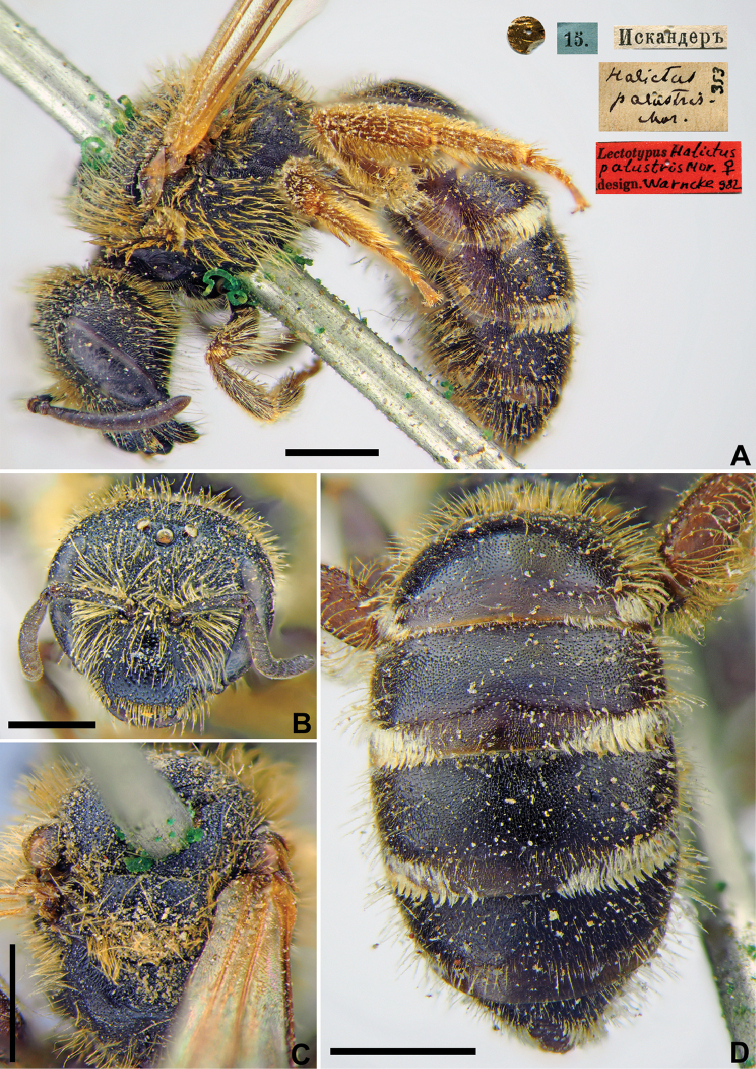
*Halictus palustris* Morawitz, 1876, lectotype, female **A** habitus, lateral view and labels **B** head, frontal view **C** mesosoma, dorsal view **D** metasoma, dorsal view. Scale bars: 1.0 mm.

##### 29. Halictus pectoralis

Taxon classificationAnimaliaHymenopteraHalictidae

Morawitz, 1876

303C41DE-B945-5FF1-877F-EFB22CE12547

Figure 24

Halictus pectoralis Morawitz, 1876: 218 (key), 251, ♀.

###### Type locality.

Gulcha (Kyrgyzstan).

###### Published (original) locality.

Kyrgyzstan: Gulsha [Gulcha].

###### Holotype.

♀, 10.[VIII.1871] // Гульша [Kyrgyzstan, Gulcha, 40°19'N, 73°26'E] // *Halictus pectoralis* Mor., [N]381 [handwritten by F. Morawitz] // Holotype *H. pectoralis* Mor., 1876 <red label, labelled by Yu. Pesenko> [ZMMU].

###### Current status.

Lasioglossum (Hemihalictus) subaenescens ssp. asiaticus (Dalla Torre, 1896), replacement name for *Halictus pectoralis* Morawitz, 1876 (nec *H. pectoralis* Smith, 1853) (subspecies status according to Ebmer 1997: 932).

###### Remarks.

Description of male. Blüthgen 1923a: 271, as *Halictus proximus* (synonymised by Warncke 1975: 96).

The lectotype designation by Warncke (1982: 106) is unnecessary as the species was described from a single female that was directly written about by Morawitz (1876: 251).

###### Distribution.

Egypt, Turkey, Near East, Iran, Azerbaijan, Central Asia, Mongolia (Hovd), China (Xinijang) (Ebmer 1997, Pesenko 2007).

**Figure 24.**
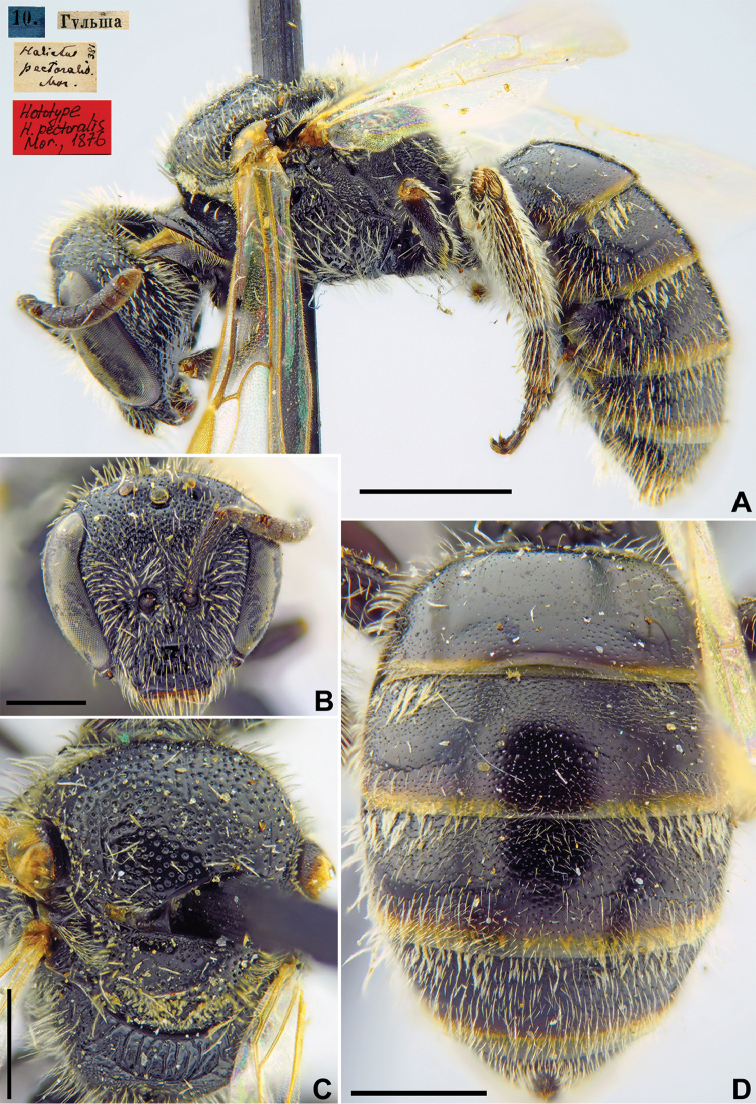
*Halictus pectoralis* Morawitz, 1876, holotype, female **A** habitus, lateral view and labels **B** head, frontal view **C** mesosoma, dorsal view **D** metasoma, dorsal view. Scale bars: 1.0 mm (**A**), 0.5 mm (**B–D**).

##### 30. Halictus picipes

Taxon classificationAnimaliaHymenopteraHalictidae

Morawitz, 1876

49D01D9B-6A20-5422-B4C4-404DB7903E8E

Figure 25

Halictus picipes Morawitz, 1876: 218 (key), 244, ♀.

###### Type locality.

Zeravshan River valley (Tajikistan).

###### Published (original) locality.

between Panjakent and Iori (Tajikistan).

###### Lectotype.

♀, designated by Pesenko 1986a: 138, 30.[V.1869] // Заравш.[анская] дол.[ина] [Tajikistan, Zeravshan River valley, between Panjakent and Iori (= Yeri)] // *Halictus picipes* Mor., [N]369 [handwritten by F. Morawitz] // Lectotype *Halictus picipes* Mor., ♀, design. Pesenko [1]985 <red label> [ZMMU].

###### Paralectotypes

(3 ♀). 1 ♀, the same label as in the lectotype [ZMMU]; 1 ♀, <golden circle>, the same label as in the lectotype; 1 ♀, Заравшан.[ская] дол.[ина] [Zeravshan River valley] // к.[оллекция] Ф. Моравица [Collection of F. Morawitz] // *Halictus picipes* Mor., ♀ [handwritten by F. Morawitz] // Paralectotypus *Hal. picipes* Mor., design. Pesenko [1]985 <identical red labels on each paralectotype specimen> [ZISP].

###### Current status.

Lasioglossum (Leuchalictus) picipes (Morawitz, 1876).

###### Remarks.

The lectotype designation by Warncke (1982: 111) is invalid because he labelled neither of the two females from “valle Serafshan” deposited in ZMMU.

Description of male. Blüthgen in Popov 1935: 362.

###### Distribution.

Israel, Turkey, Iraq, Iran, Afghanistan, Turkmenistan, Uzbekistan, Tajikistan (Pesenko 1986a, Ascher and Pickering 2020).

**Figure 25.**
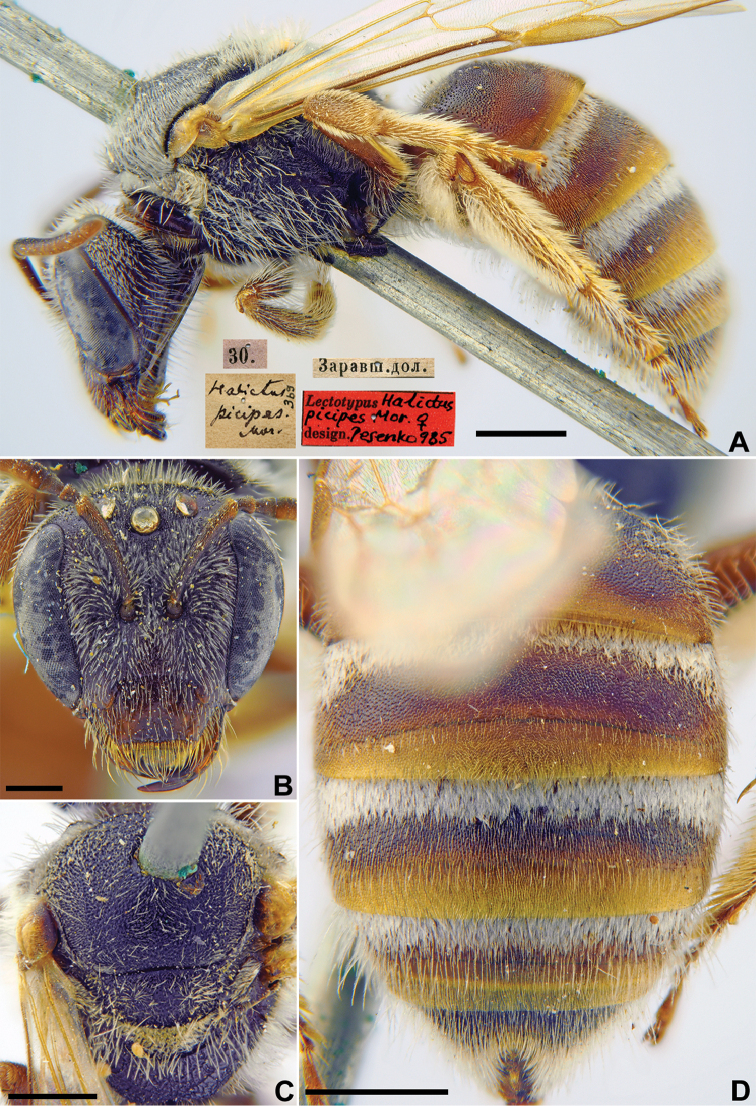
*Halictus picipes* Morawitz, 1876, lectotype, female **A** habitus, lateral view and labels **B** head, frontal view **C** mesosoma, dorsal view **D** metasoma, dorsal view. Scale bars: 1.0 mm (**A, C, D**), 0.5 mm (**B**).

##### 31. Halictus rhynchites

Taxon classificationAnimaliaHymenopteraHalictidae

Morawitz, 1876

18BB406B-2C39-53C6-93DD-036BF75BDC62

Figure 26

Halictus rhynchites Morawitz, 1876: 217 (key to females), 220 (key to males), 222, ♀, ♂.

###### Type locality.

Shakhimardan (Uzbekistan).

###### Published (original) locality.

Usbekistan: Khodzha-Chiburgan gorge, near Shakhimardan Usbekistan]; Kyrgyzstan: Alay, Kichi-alay.

###### Lectotype (designated here).

♂, 7.[VII.1871] // Шагимарданъ [Shakhimardan in the Uzbek enclave in the territory of Kyrgyzstan, Alai Ridge, 39°58'N, 71°47'E] // [N]334 // Lectotypus *Halictus rhynchites* Mor., design. Astafurova et Proshchalykin, 2020 <red label> [ZMMU].

###### Paralectotypes

(8 ♀, 5 ♂). 1 ♂, the same label as in a lectotype // *Halictus rhynchites* Mor., [N]334 [handwritten by F. Morawitz]; 1 ♀, 2 ♂, 26.[VI.1871] // Чибурганъ [Chiburgan] // [N]334; 1 ♀, 21.[VI.1871] // Чибурганъ [Chiburgan] // [N]334; 2 ♀, 28.[VII.1871] // Кичи-Алай [Kichi-Alay] // [N]334 [ZMMU]; 2 ♀, 1 ♂, Чибурганъ [Chiburgan] // к.[оллекция] Ф. Моравица [Collection of F. Morawitz] // *rhynchites* F. Mor. [handwritten by F. Morawitz]; 1 ♂, Кчи-Алай [Kchi-Alay] // к.[оллекция] Ф. Моравица [Collection of F. Morawitz] // *rhynchites* F. Mor. [handwritten by F. Morawitz]; 1 ♀, < golden circle> // 22.[VII.1871] // Алай [Alay] //*rhynchites* Mor., Typ. [handwritten by F. Morawitz]; 1 ♀, 22.[VII.1871] // Алай [Alay] // к.[оллекция] Ф. Моравица [Collection of F. Morawitz] // *Halictus rhynchites* Mor. [handwritten by F. Morawitz] // Paralectotypus *Halictus rhynchites* Mor., design. Astafurova et Proshchalykin, 2020 <identical red labels on each paralectotype specimen> [ZISP].

###### Current status.

Lasioglossum (Sphecodogastra) rhynchites (Morawitz, 1876).

###### Remarks.

The lectotype designation by Warncke (1982: 81) is invalid because he labelled neither of the two females from “Shakhimardan” deposited in ZMMU.

###### Distribution.

Turkey, Afghanistan, southern Kazakhstan, Turkmenistan, Uzbekistan, Kyrgyzstan (Ebmer 1995, Murao et al. 2017).

**Figure 26.**
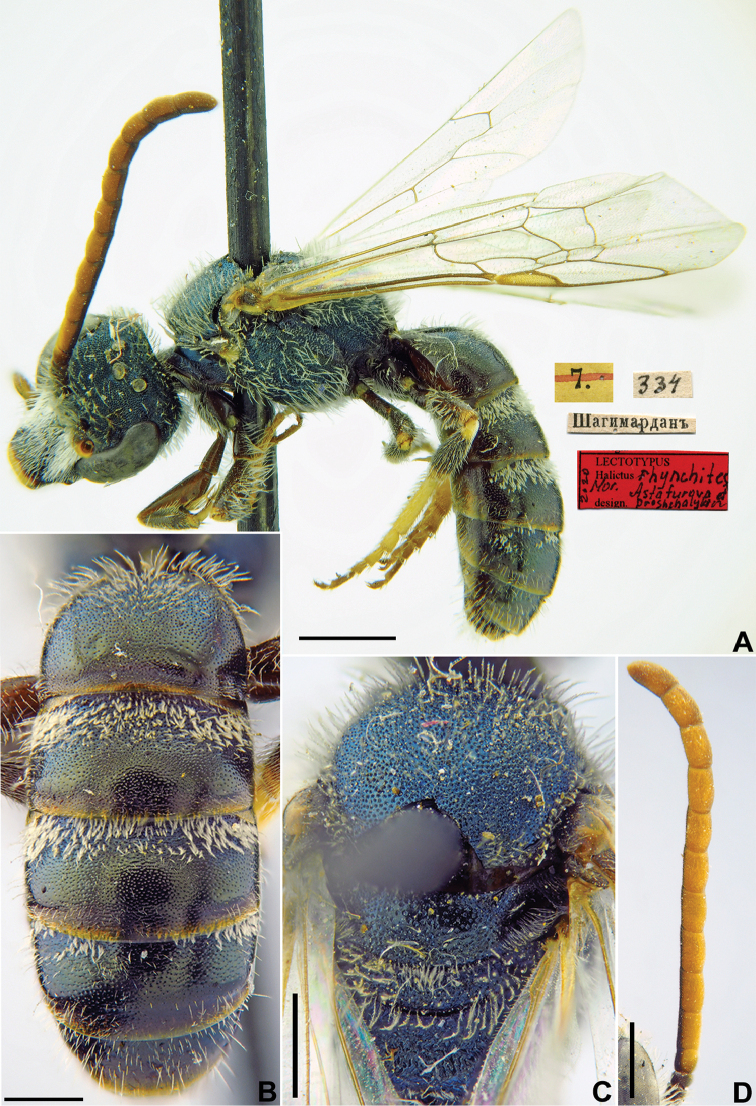
*Halictus rhynchites* Morawitz, 1876, lectotype, male **A** habitus, lateral view and labels **B** metasoma, dorsal view **C** mesosoma, dorsal view **D** antenna. Scale bars: 1.0 mm (**A**), 0.5 mm (**B–D**).

##### 32. Halictus scutellaris

Taxon classificationAnimaliaHymenopteraHalictidae

Morawitz, 1876

5F934AAA-0C72-5C79-B6AC-56423DAD631B

Figure 27

Halictus scutellaris Morawitz, 1876: 218 (key), 238, ♀.

###### Type locality.

Bairkum (Chimkent Province, Kazakhstan).

###### Published (original) locality.

Kazakhstan: Bayrakum [= Bairkum]; Tajikistan: Pendzhikent, Iori.

###### Lectotype.

♀, designated by Pesenko 1986a: 140, 4.[V.1871] // Байракумъ [Kazakhstan, Chimkent Province, Bairkum, Syr-Darya River, 42°05'N, 68°10'E] // *Halictus scutellaris* Mor., [N]359 [handwritten by F. Morawitz] // Lectotypus *Halictus scutellaris* Mor., ♀, design. Pesenko [1]985 <red label> [ZMMU].

###### Paralectotypes

(5 ♀). 1 ♀, the same label as in the lectotype; 1 ♀, 30.[V.1869] // Заравшан.[ская] дол.[ина] [Zeravshan River valley, Iori] // N[359] [ZMMU]; 1 ♀, Байракумъ [Bayrakum] // к.[оллекция] Ф. Моравица [Collection of F. Morawitz] // *scutellaris* F. Mor., ♀ [handwritten by F. Morawitz]; 2 ♀, 4.[V.1871] // Байракумъ [Bayrakum] // к.[оллекция] Ф. Моравица [Collection of F. Morawitz] // Paralectotypus *Hal. scutellaris* Mor., design. Pesenko [1]985 <identical red labels on each paralectotype specimen> [ZISP].

###### Current status.

Lasioglossum (Leuchalictus) scutellare (Morawitz, 1876).

###### Remarks.

Description of male. Blüthgen 1929: 53, as *Halictus scutellaris*.

The lectotype designation by Warncke (1982: 111) is invalid because he labelled neither of the two females from “Bayrakum” deposited in ZMMU.

###### Distribution.

Southern Kazakhstan, Turkmenistan, Tajikistan, Kyrgyzstan, China (Xinnjiang) (Pesenko 1986a, Murao et al. 2017).

**Figure 27.**
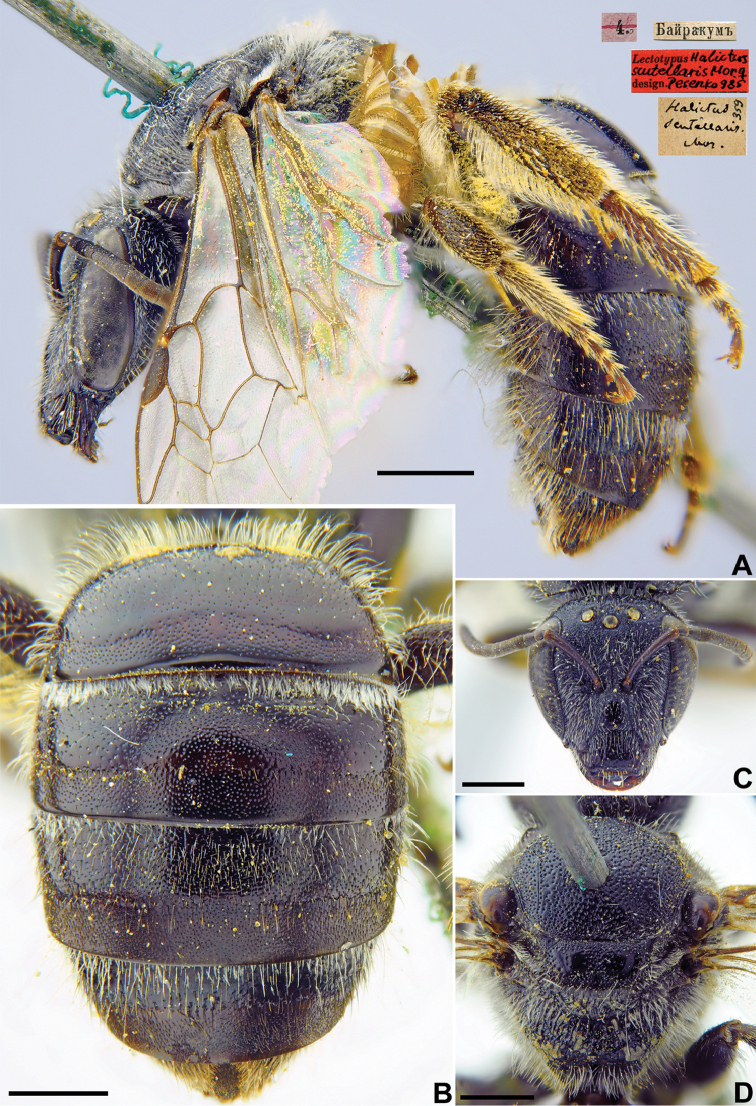
*Halictus scutellaris* Morawitz, 1876, lectotype, female **A** habitus, lateral view and labels **B** metasoma, dorsal **C** view head, frontal view **D** mesosoma, dorsal view. Scale bars: 1.0 mm.

##### 33. Halictus sogdianus

Taxon classificationAnimaliaHymenopteraHalictidae

Morawitz, 1876

534DA223-0245-5C8D-9836-BBBE2E30E5B8

Figures 28

Halictus sogdianus Morawitz, 1876: 216 (key), 227, ♀.

###### Type locality.

Samarkand (Uzbekistan).

###### Published (original) locality.

Uzbekistan: Samarkand, Dshyuzak [=Jizzakh], Iskander [River]; Kyrgyzstan: Osh.

###### Lectotype.

♀, designated by Blüthgen 1934a: 303, 7.[VII.1870] // Самаркандъ [Uzbekistan, Samarkand, 39°39'N, 66°57'E] // *Halictus sogdianus* Mor., [N]342 [handwritten by F. Morawitz] // *sogdianus* Mor., ♀, Lecto-Holotype, Blüthgen det. 1931 // Lecto-Type <red label> // Lectotypus *Halictus sogdianus* Mor., design. Blüthgen 1934 <red label> [ZMMU].

###### Paralectotypes

(5 ♀). 1 ♀, 21.[VI.1870] // Искандеръ [Iskander] // [N]342; 1 ♀, 2.[VIII. 1871] // Ошъ [Osh] // [N]342; 2 ♀, 4.[VII.1869] // Самаркандъ [Samarkand] // [N]342; 1 ♀, 18.[VII.1870] // Джюзакъ [Dzhyuzak] // [N]342 // Paralectotype *Halictus sogdianus* Mor. design. Blüthgen <identical red labels on each paralectotype specimen, labelled by Yu. Astafurova> [ZMMU].

###### Current status.

Halictus (Vestitohalictus) pulvereus Morawitz, 1874 (synonymised by Ebmer 1988b: 576).

###### Distribution.

Russia (European part, North Caucasus, Crimea), Cyprus, Turkey, Iran, Afghanistan, Central Asia, Mongolia, north-western China (Astafurova and Proshchalykin 2017).

**Figure 28.**
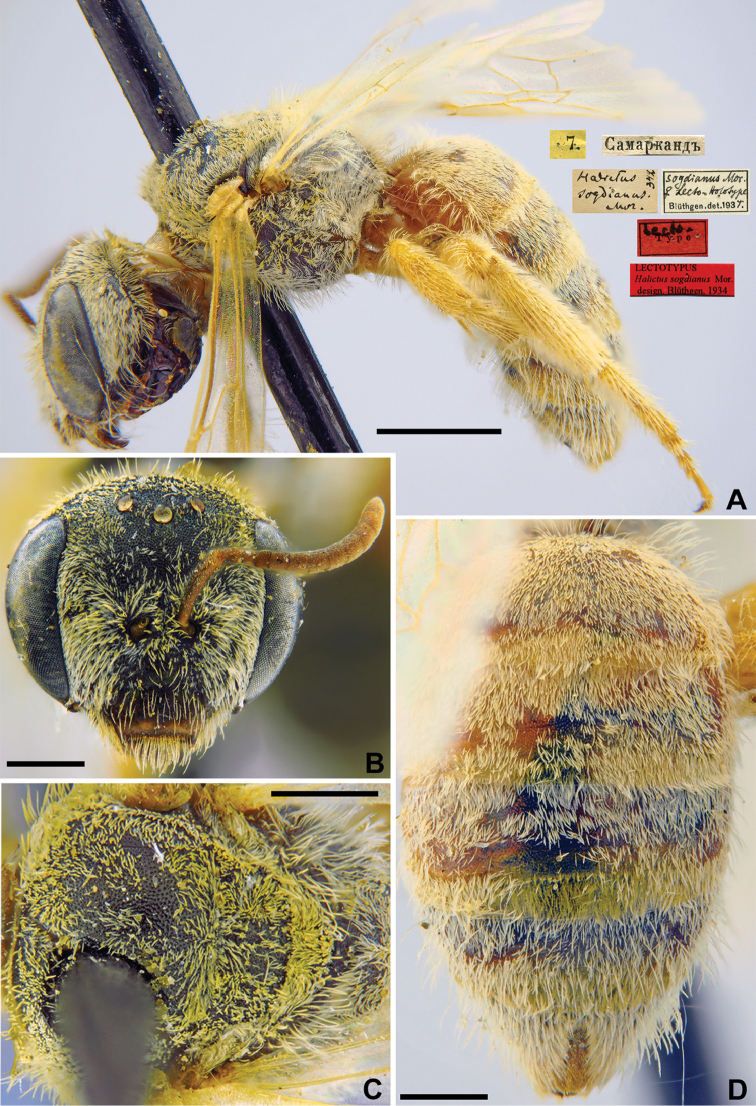
*Halictus sogdianus* Morawitz, 1876, lectotype, female **A** habitus, lateral view and labels **B** head, frontal view **C** metasoma, dorsal view **D** mesosoma, dorsal view. Scale bars: 1.0 mm (**A**), 0.5 mm (**B–D**).

##### 34. Halictus trifasciatus

Taxon classificationAnimaliaHymenopteraHalictidae

Morawitz, 1876

FDA0BA5F-415E-5762-AF30-281E7AD30236

Figure 29

Halictus trifasciatus Morawitz, 1876: 218 (key), 240, ♀.

###### Type locality.

Bairkum (Chimkent Province, Kazakhstan).

###### Published (original) locality.

Kazakhstan: Bayrakum.

###### Lectotype.

♀, designated by Warncke 1982: 90, <golden circle> // 4.[V.1871] // Байракумъ [Kazakhstan, Chimkent Province, Bairkum, Syr-Darya River, 42°05'N, 68°10'E] // *Halictus trifasciatus* Mor., [N]362 [handwritten by F. Morawitz] // Lectotypus *Halictus trifasciatus* Mor., design. Warncke [19]82 <red label, labelled by Yu. Pesenko> [ZMMU].

###### Current status.

Lasioglossum (Lasioglossum) lebedevi Ebmer, 1972, replacement name for *Halictus trifasciatus* Morawitz, 1876 (nec *Hylaeus trifasciatus* Schenck, 1853).

###### Remarks.

Male unknown.

###### Distribution.

Southern Kazakhstan (Morawitz 1876, Mutao et al. 2017). The record from Azerbaijan (Aliyev et al. 2007) is doubtful and needs checking.

**Figure 29.**
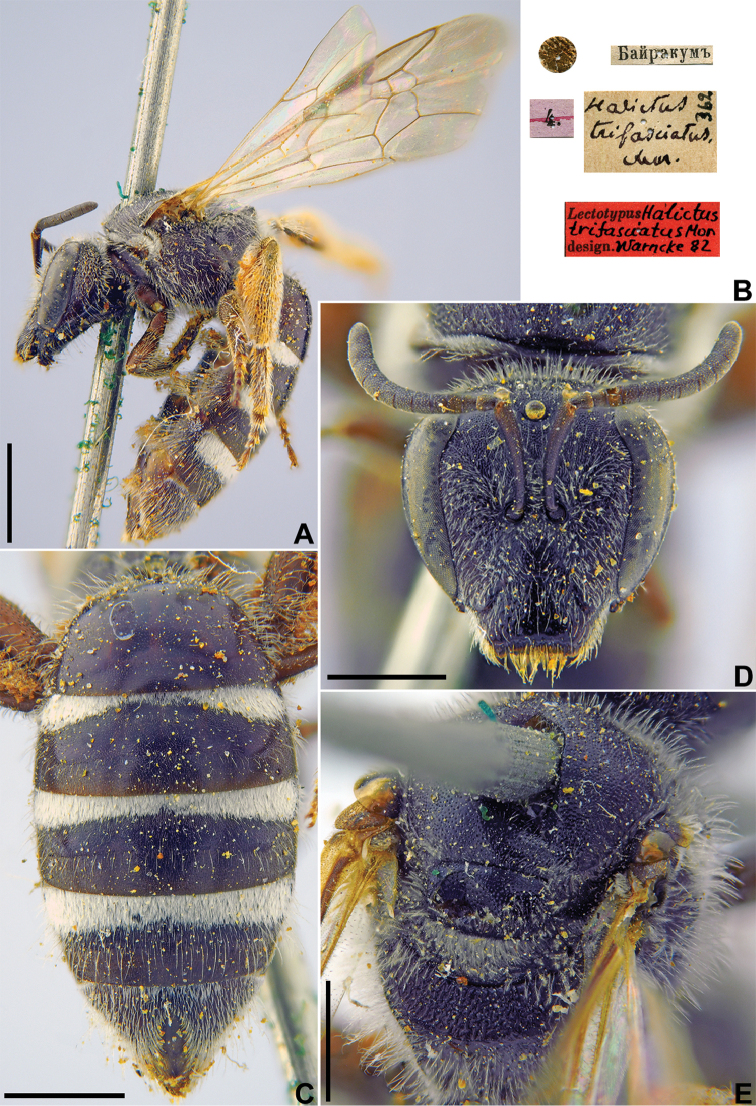
*Halictus trifasciatus* Morawitz, 1876, lectotype, female **A** habitus, lateral view **B** labels **C** metasoma, dorsal view **D** head, frontal view **E** mesosoma, dorsal view. Scale bars: 2.0 mm (**A**), 1.0 mm (**C–E**).

##### 35. Halictus varipes

Taxon classificationAnimaliaHymenopteraHalictidae

Morawitz, 1876

06CD3366-2926-552D-8046-EEA89F2366B4

Figure 30

Halictus varipes Morawitz, 1876: 217 (key to females), 220 (key to males), 223, ♀, ♂.

###### Type locality.

Jizzakh (Uzbekistan).

###### Published (original) locality.

Uzbekistan: Katty-Kurgan [= Kattakurgan], Dzhyuzak [= Jizzakh], Karatyube [= Karatepa near Samarkand], Urgut, Sangy-Dzhuman; Kyrgyzstan: near Osh.

###### Lectotype.

♀, designated by Blüthgen 1955: 17, 19.[VII.1870] // Джюзакъ [Uzbekistan, Dzhyuzak (= Jizzakh), 40°07'N, 67°51'E] // [N]337 // *Halictus varipes* Mor., ♀, Lecto-Holotype, P. Blüthgen det. // Typus <red label> // Lectotypus *Halictus varipes* Mor., 1876, design. Blüthgen, 1955 <red label> [ZMMU].

###### Paralectotypes

(1 ♀, 2 ♂). 1 ♂, the same labels as in the lectotype // *Halictus varipes* Mor. ♂, lecto-Paratype, Blüthgen det.; 1 ♂, the same label, but 14.[VII.1870] // *Halictus varipes* Mor. ♂, lecto-Holotype, Blüthgen det.; 1 ♀, 20. [VI.1869] // Катты-Курганъ [Katty-Kurgan] // *Halictus varipes* Mor. ♀, lecto-Paratype, Blüthgen det. // Paralectotype *Halictus varipes* Mor., design. Blüthgen 1955 <identical red labels on each paralectotype specimen, labelled by Yu. Astafurova> [ZMMU].

###### Current status.

Halictus (Seladonia) lucidipennis Smith, 1853 (synonymised by Sakagami and Ebmer 1987: 326).

###### Distribution.

Southern Palaearctic and Oriental Regions. North Africa, Israel, Arabian Peninsula, Turkey, Central Asia, Iraq, Iran, Afghanistan, Pakistan, Mongolia, north-western China, India, Nepal, Myanma,Thailand, Sri Lanka (Pesenko 2006a).

**Figure 30.**
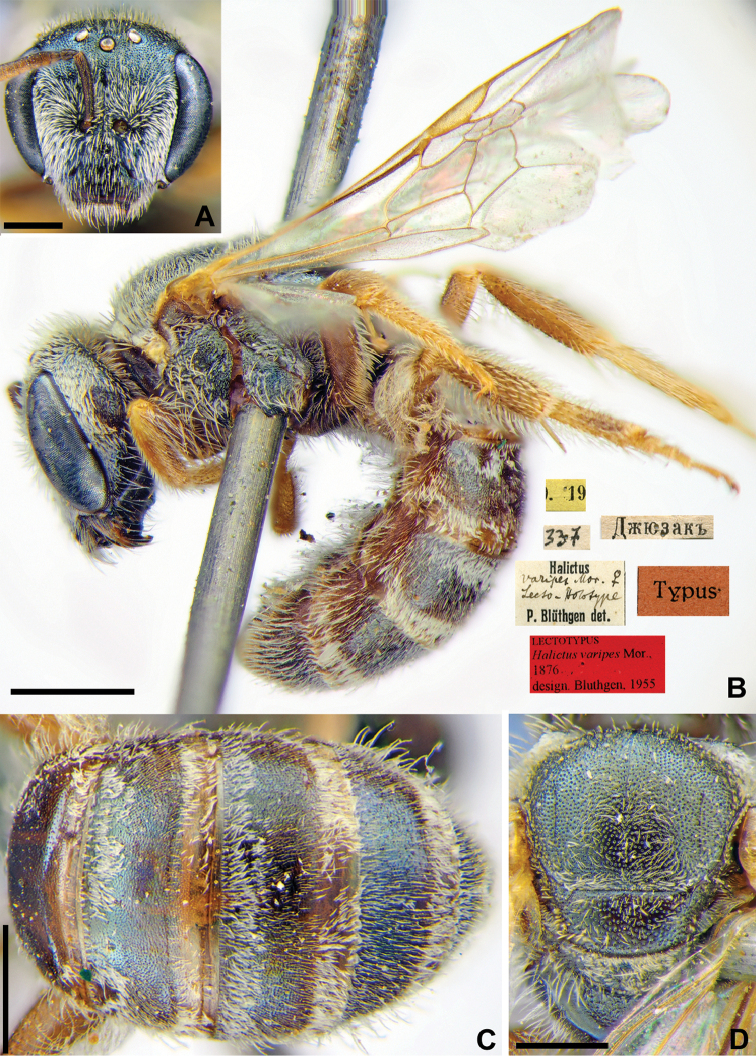
*Halictus varipes* Morawitz, 1876, lectotype, female **A** head, frontal view **B** habitus, lateral view and labels **C** metasoma, dorsal view **D** mesosoma, dorsal view. Scale bars: 1.0 mm (**B**), 0.5 mm (**A, C, D**).

##### 36. Halictus vulgaris

Taxon classificationAnimaliaHymenopteraHalictidae

Morawitz, 1876

FD313813-96D6-5A74-A2FE-EE46362F4513

Figure 31

Halictus vulgaris Morawitz, 1876: 218 (key), 250, ♀.

###### Type locality.

Samarkand (Uzbekistan).

###### Published (original) locality.

Uzbekistan: Tashkent, Samarkand, Katty-Kurgan [Kattakurgan].

###### Lectotype (designated here).

♀, 3.[III.1869] [Uzbekistan, Samarkand, 39°39'N, 66°57'E// *Hylaeus* [sic!] *vulgaris* Mor., [N]379 [handwritten by F. Morawitz] // Lectotypus *Halictus vulgaris* Mor., design. Astafurova et Proshchalykin, 2020 <red label> [ZMMU].

###### Paralectotypes

(257 ♀). 26 ♀, 4., 20., 23., 30.[III.1869], 3., 11., 19.[IV.1869] // Самаркндъ [Samarkand]; 5 ♀, 28.[IV.1869] // Каттыкурганъ [Kattykurgan]; 222♀, 10., 11., 24., 26., 27., 28., [II.1871], 23., 24. [III.1871], 1., 2., 3., 5., 8., 10., 11.[IV.1871] // Ташкентъ [Tashkent] [ZMMU]; 1 ♀, 3.[IV.1871] // Ташкентъ [Tashkent] // к. Ф. Моравица // *Halictus vulgaris* Mor. [handwritten by F. Morawitz]; 1 ♀, 5.[IV.1871] // Ташкентъ [Tashkent] // к.[оллекция] Ф. Моравица [Collection of F. Morawitz]; 2 ♀, 8.[IV.1871] // Ташкентъ [Tashkent] // к.[оллекция] Ф. Моравица [Collection of F. Morawitz]; 1 ♀, 1.[IV.1871] // Ташкентъ [Tashkent] // к.[оллекция] Ф. Моравица [Collection of F. Morawitz] // Paralectotypus, *Halictus vulgaris* Mor., design. Astafurova et Proshchalykin <identical red labels on each paralectotype specimen> [ZISP].

###### Current status.

Lasioglossum (Evylaeus) marginatum (Brullé, 1832) (synonymised by Blüthgen 1926: 391).

###### Distribution.

Europe (except North), North Africa, Caucasus, Russia (East of European part, North Caucasus), Turkey, Syria, Jordan, Israel, Iraq, Iran, Afghanistan, Pakistan, Central Asia, Kazakhstan, north India, Nepal (Astafurova and Proshchalykin 2017).

###### Remarks.

The lectotype designation by Warncke (1982: 116) is invalid because he labelled none of the 222 females from Tashkent deposited in ZMMU.

**Figure 31.**
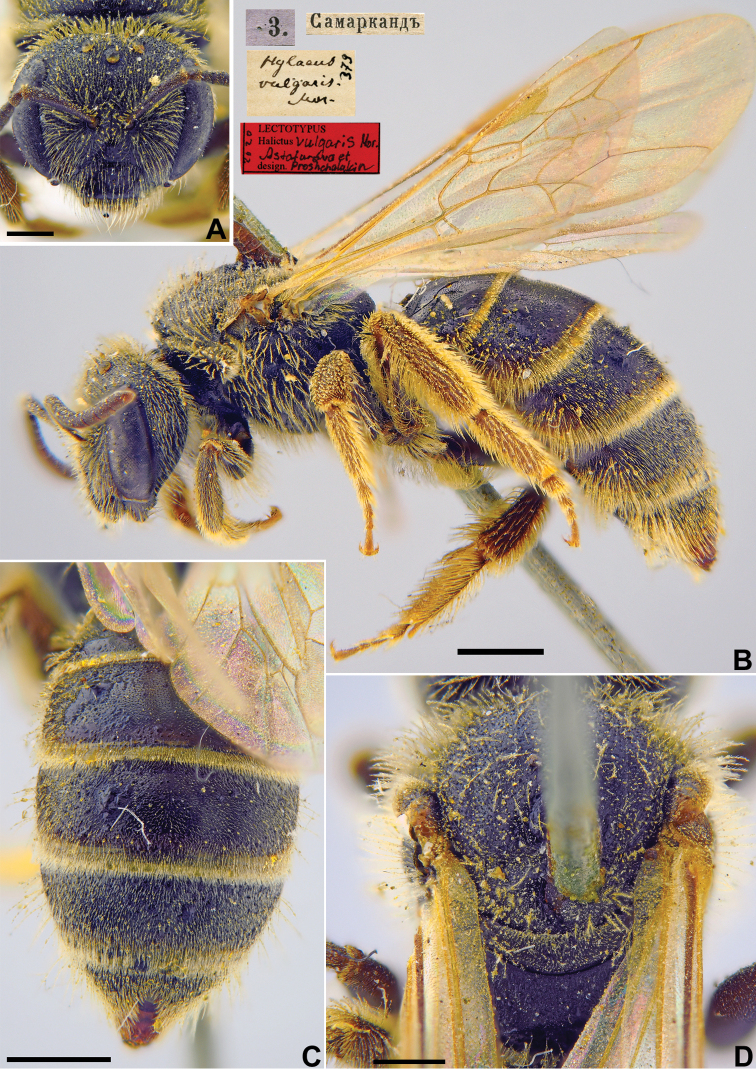
*Halictus vulgaris* Morawitz, 1876, lectotype, female **A** head, frontal view **B** habitus, lateral view and labels **C** metasoma, dorsal view **D** mesosoma, dorsal view. Scale bars: 1.0 mm (**B, C**), 0.5 mm (**A, D**).

#### Genus *Sphecodes* Latreille, 1804

##### 37. Sphecodes nigripennis

Taxon classificationAnimaliaHymenopteraHalictidae

Morawitz, 1876

2E2C1C94-0E05-5CEF-86BE-FD06A326124C

Figure 32

Sphecodes nigripennis Morawitz, 1876: 257, ♀.

###### Type locality.

Shardara District of Turkistan Province (Kazakhstan).

###### Published (original) locality.

Kazakhstan: coasts of Kosaral Lake; Uzbekistan: Khodzhaduk [= Khozyay-Dun].

###### Lectotype.

♀, designation by Warncke 1992: 30, 24.IV.1871 [the original blue data label was damaged] // Косаралъ [Kazakhstan, “Kosaral Lake”, Shardara (= Chardara) District of Turkistan (= South-Kazakhstan) Province, ≈ 41°10'N, 68°06'E] // *Sphecodes nigripennis* Mor. [handwritten by F. Morawitz] // Lectotypus, Warncke 1975 <red label> [ZMMU].

###### Paralectotype.

1 ♀, 21.[V.1869] // Заравшан.[ская] дол.[ина] [Zeravshan River valley] // Paralectotype *Sphecodes nigripennis* Mor., design. Warncke <red label, labelled by Yu. Astafurova> [ZMMU].

###### Current status.

*Sphecodes gibbus* (Linnaeus 1758) (synonymised by Blüthgen 1923b: 510).

###### Distribution.

North Africa, Europe (north to 63°), Israel, Jordan, Russia (east to Yakutia), Turkey, Iran, Pakistan, Central Asia, Kazakhstan, Mongolia, NW China, India (Warncke 1992, Bogusch and Straka 2012, Astafurova et al. 2019).

**Figure 32.**
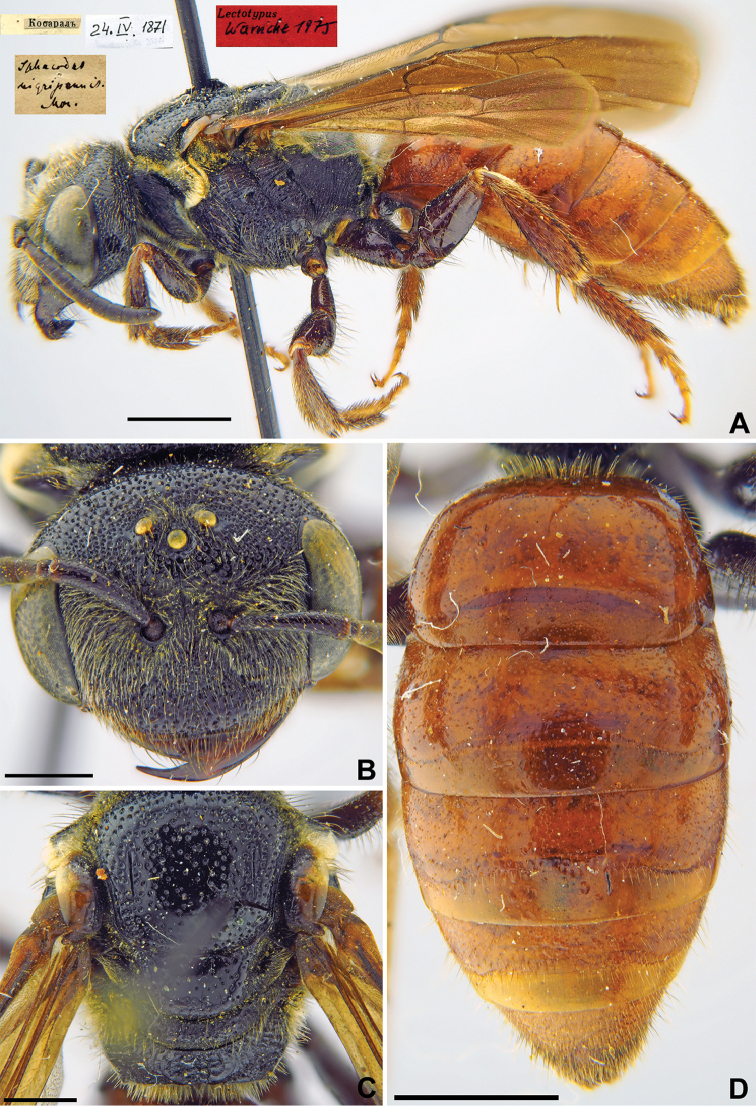
*Sphecodes nigripennis* Morawitz, 1876, lectotype, female **A** habitus, lateral view and labels **B** head, frontal view **C** mesosoma, dorsal view **D** metasoma, dorsal view. Scale bars: 2.0 mm (**A, D**), 1.0 mm (**B, C**).

##### 38. Sphecodes pectoralis

Taxon classificationAnimaliaHymenopteraHalictidae

Morawitz, 1876

168597F0-F09A-5EC4-B44F-55A8A2EA0C4E

Figure 33

Sphecodes pectoralis Morawitz, 1876: 256, ♀.

###### Type locality.

Shardara District of Turkistan Province (Kazakhstan).

###### Published (original) locality.

Kazakhstan: coasts of Kosaral Lake; Kyzylkum [desert] near Chakany Well.

###### Lectotype.

♀, designation by Warncke 1992: 24, 24.[IV.1871] // Косаралъ [Kazakhstan, “Kosaral Lake”, Shardara (= Chardara) District of Turkistan (= South-Kazakhstan) Province], ≈ 41°10'N, 68°06'E// *Sphecodes pectoralis* Mor. [handwritten by F. Morawitz] // Lectotypus, Warncke 1975 <red label> [ZMMU].

###### Paralectotype.

1 ♀, 28.[VI.1871] // Кызылъкумъ [Kyzylkum] // Paralectotype *Sphecodes pectoralis* Mor., design. Warncke <red label, labelled by Yu. Astafurova> [ZMMU].

###### Current status.

*Sphecodes pectoralis* Morawitz, 1876.

###### Remarks.

Description of male. Meyer 1919: 126, as *Sphecodes cristatus* sensu Meyer (non Hagens 1882) (see Blüthgen 1924: 475).

###### Distribution.

South Kazakhstan, Central Asia, China (Gansu, Xinjiang) (Astafurova et al. 2018a, b, 2020).

**Figure 33.**
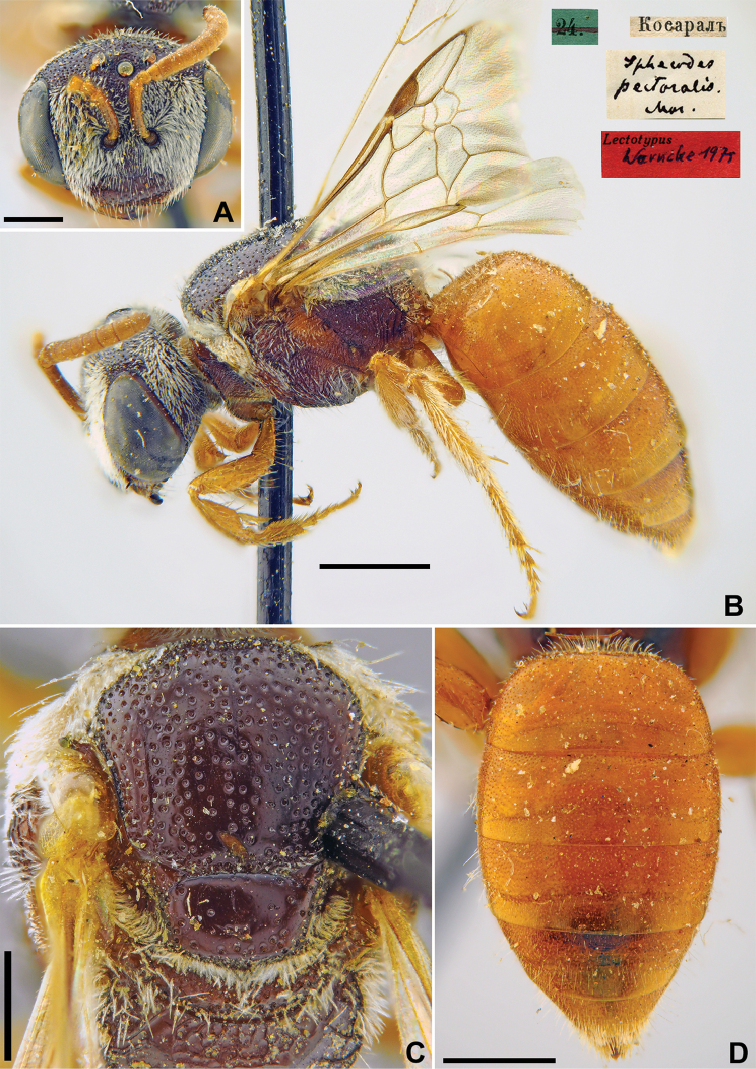
*Sphecodes pectoralis* Morawitz, 1876, lectotype, female **A** head, frontal view **B** habitus, lateral view and labels **C** mesosoma, dorsal view **D** metasoma, dorsal view. Scale bars: 2.0 mm (**B, D**), 1.0 mm (**A, C**).

##### 39. Sphecodes rufithorax

Taxon classificationAnimaliaHymenopteraHalictidae

Morawitz, 1876

871E77F6-9DDB-5017-9259-074246B9574D

Figure 34

Sphecodes rufithorax Morawitz, 1876: 255, ♀, ♂.

###### Type locality.

Bairkum (Chimkent Province, Kazakhstan).

###### Published (original) locality.

Kazakhstan: Bayrakum [Bairkum]; steppe between Syr-Darya River and Tashkent.

###### Lectotype.

♀, designated by Warncke 1992: 24, 17. [V.1871] // Байракумъ [Kazakhstan, Chimkent Province, Bairkum, Syr-Darya River, 42°05'N, 68°10'E] // *Sphecodes rufithorax* F. Moraw., ♀ [handwritten by F. Morawitz] // Lectotypus Warncke, 1975 <red label> [ZMMU].

###### Paralectotypes

(2 ♀, 1 ♂). 1 ♀, Байракумъ [Bairkum] // кол.[лекция] Ф. Моравица [Collection of F. Morawitz] // *Sphecodes rufithorax* F. Moraw., ♀ [handwritten by F. Morawitz] // F. Morawitz det., Typ.; 1 ♀, 20.[V.1871] // Степь м.[ежду] С.[ыр] д.[арьей] и Т.[ашкентом] [Steppe between Syrdarya River and Tashkent] // *Sphecodes rufithorax* Mor., ♂ [handwritten by F. Morawitz] // Paralectotypus *Sphec. rufithorax* Mor., design. Warncke, [19]92 <red label, labelled by Yu. Astafurova> [ZMMU]; 1 ♀, the same labels [ZISP].

###### Current status.

*Sphecodes olivieri* Lepeletier, 1825 (synonymised by Warncke 1992: 24).

###### Distribution.

North Africa, the Arabian Peninsula, Israel, Jordan, South Europe, Russia (south of European part), Turkey, Caucasus, Iran, Pakistan, Central Asia, Kazakhstan, NW China (Astafurova et al. 2019).

**Figure 34.**
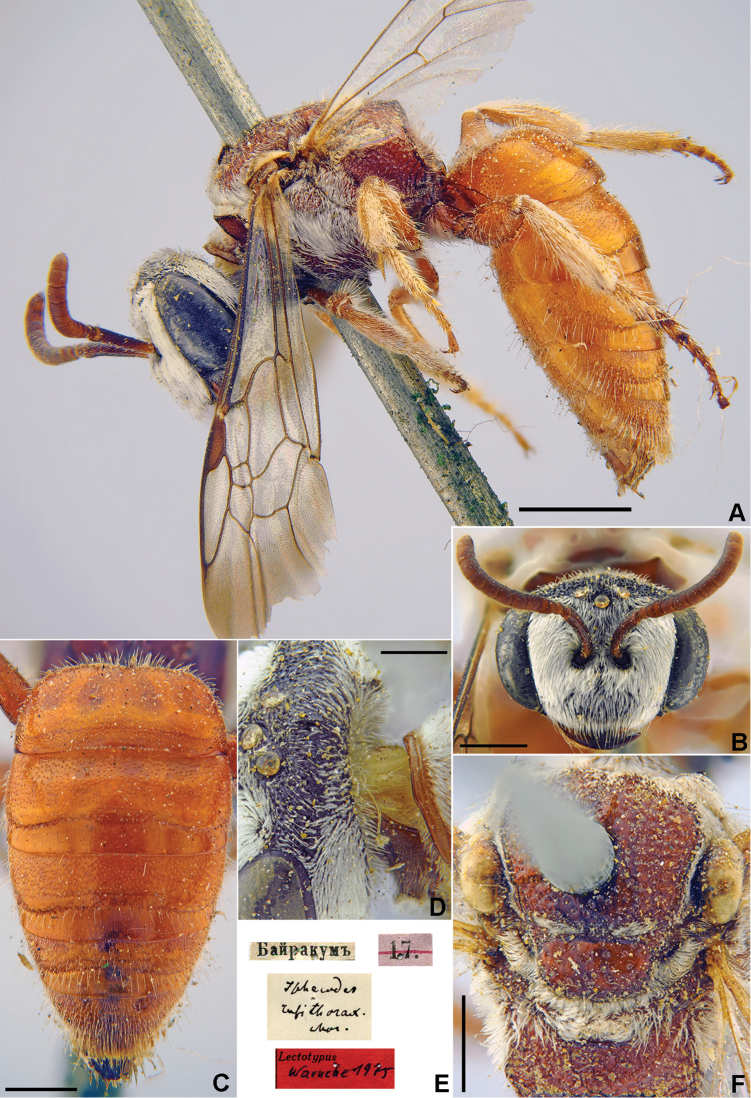
*Sphecodes rufithorax* Morawitz, 1876, lectotype, female **A** habitus, lateral view **B** head, frontal view **C** metasoma, dorsal view **D** vertex, dorso-lateral view **E** labels **F** mesosoma, dorsal view. Scale bars: 1.0 mm (**A–C, F**), 0.5 mm (**D**).

#### Subfamily Nomiinae

**Genus *Nomia* Latreille, 1804**

##### 40. Nomia edentata

Taxon classificationAnimaliaHymenopteraHalictidae

Morawitz, 1876

D6C3CB5F-DCAE-5E9E-8BD0-AAAB4FB031EA

Figure 35

Nomia edentata Morawitz, 1876: 259, ♀, ♂.

###### Type locality.

Jizzakh (Uzbekistan).

###### Published (original) locality.

Uzbekistan: Samarkand, Dzhyuzak.

###### Lectotype.

♀, designated by Warncke 1976: 104, 20.[VII.1871] // Джюзакъ [Uzbekistan, Dzhyuzak (= Jizzakh), 40°07'N, 67°51'E] //*Nomia edentata* Mor. [handwritten by F. Morawitz] // Lectotypus, Warncke 1975 <red label> [ZMMU].

###### Paralectotypes

(2 ♂). 1 ♂, the same labels as in the lectotype // Paralectotypus, *Nomia edentata* Mor., design. Warncke <red label> [ZISP]; 1 ♂, 8.[VII.1869] // Самаркандъ [Samarkand] // Paralectotypus *Nomia edentata* Mor., design. Warncke <red label> [ZMMU].

###### Current status.

*Pseudapis edentata* (Morawitz, 1876).

###### Distribution.

North Africa, Saudi Arabia, Turkey, Azerbaijan, Kazakhstan, Central Asia, Iraq, Iran, Afghanistan, Pakistan, India (Astafurova 2014).

**Figure 35.**
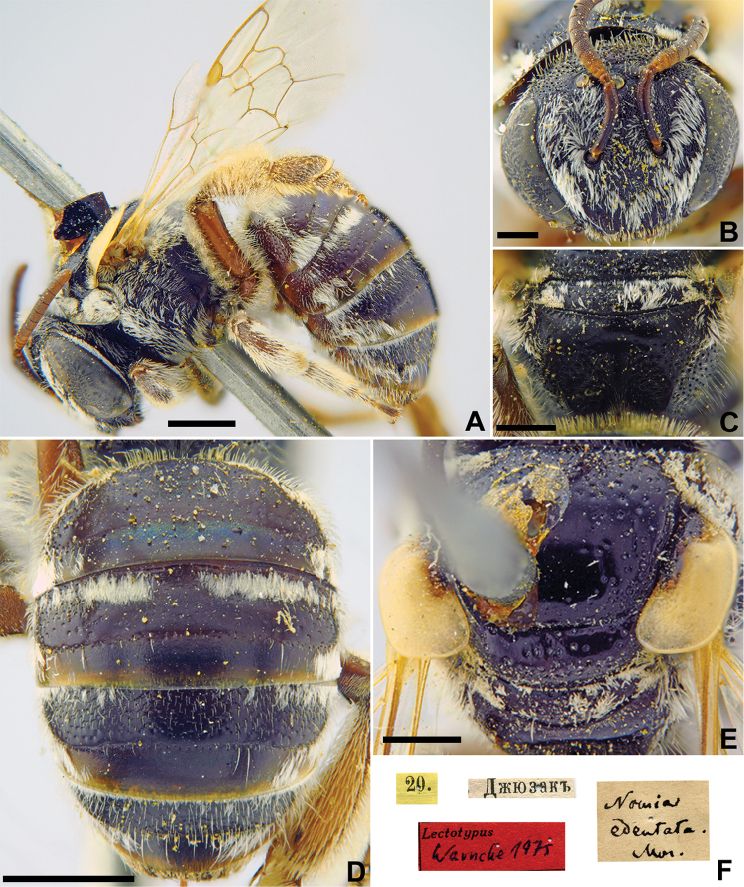
*Nomia edentata* Morawitz, 1876, lectotype, female **A** habitus, lateral view **B** head, frontal view **C** metapostnotum, dorsal view **D** metasoma, dorsal view **E** mesosoma, dorsal view **F** labels. Scale bars: 1.0 mm (**A, D**), 0.5 mm (**B, C, E**).

##### 41. Nomia rufescens

Taxon classificationAnimaliaHymenopteraHalictidae

Morawitz, 1876

65869E10-D5BC-5910-AE38-7623BFCCFE47

Figure 36

Nomia rufescens Morawitz, 1876: 261, ♀.

###### Type locality.

Zeravshan River valley (Uzbekistan).

###### Published (original) locality.

Uzbekistan: “Aykul Lake” in Zeravshan River valley.

###### Lectotype.

♀, designated by Warncke 1976: 106, 5.[VIII.1869] // Заравш[анская]. дол.[ина] [Uzbekistan, Zeravshan River valley, Aykul Lake near Chelek, 39°56'N, 66°49'E] // *Nomia rufescens* Mor. // Lectotypus, Warncke 1975 <red label> [ZMMU].

###### Current status.

*Pseudapis rufescens* (Morawitz, 1876).

###### Remarks.

Description of male. Morawitz 1893: 79.

###### Distribution.

Turkey, Central Asia, Kazakhstan (Astafurova 2014).

**Figure 36.**
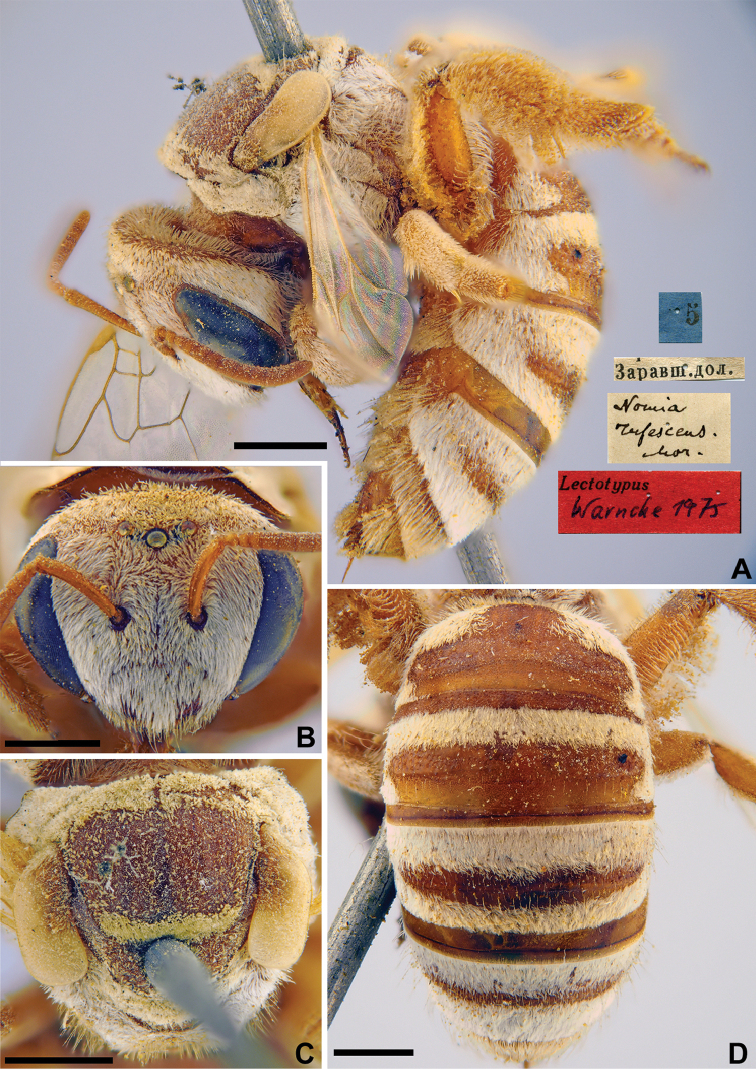
*Nomia rufescens* Morawitz, 1876, lectotype, female **A** habitus, lateral view and labels **B** head, frontal view **C** mesosoma, dorsal view **D** metasoma, dorsal view. Scale bars: 1.0 mm.

#### Subfamily Nomioidinae

**Genus *Nomioides* Schenck, 1866**

##### 42. Nomioides parviceps

Taxon classificationAnimaliaHymenopteraHalictidae

Morawitz, 1876

A04B3F23-E067-5F3E-8AE2-0316930DAE54

Figure (see Astafurova and Proshchalykin 2019: 56, figs 51a–e)

Nomioides parviceps Morawitz, 1876: 215, ♂.

###### Type locality.

Bairamali (Turkmenistan).

###### Published (original) locality.

Uzbekistan: Samarkand.

###### Holotype.

♂, окр.[естности] Самарканда [Uzbekistan, Samarkand, 39°39'N, 66°57'E], 13.VI[1869], lost (also see Pesenko 1983: 168–170).

###### Neotype.

♂, designated by Pesenko 1983: 168, Байрам-Али Закасп.[ийcкая] обл.[асть] [Turkmenistan, Maryi Province, Bairamali, 37°37'N, 62°09'E], 14.VII.[1]928, В. Гуссаковский [V. Gussakovskij leg.] // *Nomioides conjungens* m., ♂, Blüthgen det. 1931 // Neotypus *Nomioides parviceps* Mor., ♂, design. Pesenko [1]980 [handwritten by Yu. Pesenko] <red label> // Zoological Institute St. Petersburg, INS_HYM_0000142 [ZISP].

###### Current status.

Nomioides (Nomioides) parviceps Morawitz, 1876.

###### Remarks.

Description of female: Blüthgen 1925b: 45, as *Nomioides conjungens* (synonymised by Blüthgen 1934c: 253).

###### Distribution.

Asia Minor, Afghanistan, Armenia, Central Asia (Astafurova and Proshchalykin 2019).

##### 43. Nomioides turanica

Taxon classificationAnimaliaHymenopteraHalictidae

Morawitz, 1876

12CF6959-1988-5CDD-8935-CB84BD38C483

Figure (see Astafurova and Proshchalykin 2019: 68, figs 62a–e).

Nomioides turanica Morawitz, 1876: 214, ♀, ♂.

###### Type locality.

Samarkand (Uzbekistan).

###### Published (original) locality.

Tajikistan: Murzarabat; Uzbekistan: Sokh, Samarkand.

###### Lectotype.

♂, designated by Pesenko 1983: 174, 5.[VII.1870] // Самаркандъ [Uzbekistan, Samarkand, 39°39'N, 66°57'E] // *turanica* Mor., Typ. [handwritten by F. Morawitz] // Lectotypus *Nom. turanica* Mor., ♂, design. Pesenko, 1976 // Zoological Institute St. Petersburg, INS_HYM_0000131 [ZISP].

###### Paralectotypes

(5 ♂). 1 ♂, 28.[VI.1871] // Сохъ [Sokh] // *Nomioides turanica* n. sp. F. Morawitz det.; 1 ♂, <gold circle> 8.[VII.1870] // Самаркандъ [Samarkand] // *Nomioides turanica* Mor. [handwritten by F. Morawitz] [ZMMU]; 3 ♂, Сохъ [Sokh] // к.[оллекция] Ф. Моравица [Collection of F. Morawitz] // *Nomioides turanica* Mor. // Paralectotypus ♂ *Nom. turanica* Mor., design. Pesenko, 1976 <identical red labels on each paralectotype specimen> [ZISP].

###### Current status.

Nomioides (Nomioides) turanicus Morawitz, 1876.

###### Distribution.

North Africa, Central Asia, Iran, Pakistan (Astafurova and Proshchalykin 2019).

## Supplementary Material

XML Treatment for Halictus albitarsis

XML Treatment for Halictus annulipes

XML Treatment for Halictus aprilinus

XML Treatment for Halictus atomarius

XML Treatment for Halictus cariniventris

XML Treatment for Halictus cingulatus

XML Treatment for Halictus croceipes

XML Treatment for Halictus desertorum

XML Treatment for Halictus determinatus

XML Treatment for Halictus equestris

XML Treatment for Halictus ferghanicus

XML Treatment for Halictus fucosus

XML Treatment for Halictus fulvitarsis

XML Treatment for Halictus funerarius

XML Treatment for Halictus fuscicollis

XML Treatment for Halictus hyalinipennis

XML Treatment for Halictus laevinodis

XML Treatment for Halictus limbellus

XML Treatment for Halictus longirostris

XML Treatment for Halictus maculipes

XML Treatment for Halictus melanarius

XML Treatment for Halictus minor

XML Treatment for Halictus modernus

XML Treatment for Halictus nasica

XML Treatment for Halictus nigrilabris

XML Treatment for Halictus nigripes

XML Treatment for Halictus obscuratus

XML Treatment for Halictus palustris

XML Treatment for Halictus pectoralis

XML Treatment for Halictus picipes

XML Treatment for Halictus rhynchites

XML Treatment for Halictus scutellaris

XML Treatment for Halictus sogdianus

XML Treatment for Halictus trifasciatus

XML Treatment for Halictus varipes

XML Treatment for Halictus vulgaris

XML Treatment for Sphecodes nigripennis

XML Treatment for Sphecodes pectoralis

XML Treatment for Sphecodes rufithorax

XML Treatment for Nomia edentata

XML Treatment for Nomia rufescens

XML Treatment for Nomioides parviceps

XML Treatment for Nomioides turanica
